# Chemistry of Renieramycins. Part 19: Semi-Syntheses of 22-*O*-Amino Ester and Hydroquinone 5-*O*-Amino Ester Derivatives of Renieramycin M and Their Cytotoxicity against Non-Small-Cell Lung Cancer Cell Lines

**DOI:** 10.3390/md18080418

**Published:** 2020-08-10

**Authors:** Supakarn Chamni, Natchanun Sirimangkalakitti, Pithi Chanvorachote, Khanit Suwanborirux, Naoki Saito

**Affiliations:** 1 Natural Products and Nanoparticles Research Unit (NP^2^), Chulalongkorn University, Bangkok 10330, Thailand; 2 Department of Pharmacognosy and Pharmaceutical Botany, Faculty of Pharmaceutical Sciences, Chulalongkorn University, Pathumwan, Bangkok 10330, Thailand; Khanit.S@pharm.chula.ac.th; 3 Graduate School of Pharmaceutical Sciences, Meiji Pharmaceutical University, 2-522-1 Noshio, Kiyose, Tokyo 204-8588, Japan; kikns@hotmail.com; 4 Department of Pharmacology and Physiology, Faculty of Pharmaceutical Sciences, Chulalongkorn University, Pathumwan, Bangkok 10330, Thailand; pithi_chan@yahoo.com

**Keywords:** renieramycin M, bistetrahydroisoquinolinequinone, marine alkaloid, 22-*O*-amino ester derivatives of renieramycin M, hydroquinone 5-*O*-amino ester derivatives of renieramycin M, semi-synthesis, chemical modification, non-small-cell lung cancer, cytotoxicity

## Abstract

Two new series of synthetic renieramycins including 22-*O*-amino ester and hydroquinone 5-*O*-amino ester derivatives of renieramycin M were semi-synthesized and evaluated for their cytotoxicity against the metastatic non-small-cell lung cancer H292 and H460 cell lines. Interestingly, the series of 22-*O*-amino ester derivatives displayed a potent cytotoxic activity greater than the hydroquinone derivatives. The most cytotoxic derivative of the series was the 22-*O*-(*N*-Boc-l-glycine) ester of renieramycin M (**5a**: IC_50_ 3.56 nM), which showed 7-fold higher potency than renieramycin M (IC_50_ 24.56 nM) and 61-fold more than jorunnamycin A (IC_50_ 217.43 nM) against H292 cells. In addition, **5a** exhibited a significantly higher cytotoxic activity than doxorubicin (ca. 100 times). The new semi-synthetic renieramycin derivatives will be further studied and developed as potential cytotoxic agents for non-small-cell lung cancer treatment.

## 1. Introduction

Marine natural products have been documented as fascinating biologically active compounds. According to their structural complexity, marine molecules are capable of interacting with numerous biomolecular targets in the living cells to either inhibit or promote specific biological functions for therapeutic purposes [1]. Regarding the rich biodiversity of ocean creatures, several series of new natural products have been isolated from different marine organisms and reported as potential anti-cancer agents [2,3,4]. Several marine compounds and their synthetic derivatives are currently being investigated in different clinical trials [5,6]. To date, six marine-derived drugs have been approved by the United States Food and Drug Administration (U.S. FDA) including four anticancer drugs: cytarabine, eribulin mesylate, brentuximab vedotin, and trabectedin [7,8].

Various known marine and microbial alkaloids belong to the tetrahydroisoquinoline family such as renieramycins, ecteinascidins, saframycins, safracins, naphthyridinomycins, and jorunnamycins. Interestingly, 1,2,3,4-tetrahydroisoquinoline analogs possess attractive anti-cancer activity against several cancer cell lines including leukemia, lung, and colon [9,10,11,12]. The 1,2,3,4-tetrahydroisoquinoline motif played an essential role as a minor groove DNA alkylator that covalently bonds specifically to the N2 position of guanine, which led to DNA bending toward the major groove and caused DNA damage in cancer cells [13]. These results have emphasized the importance of this naturally occurring 1,2,3,4-tetrahydroisoquinoline scaffold as a promising template for anti-cancer candidates.

**Figure 1:**
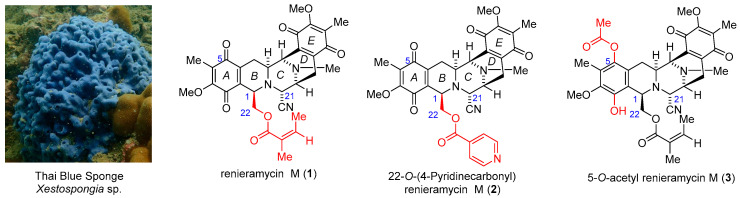
The Thai blue sponge *Xestospongia* sp., renieramycin M (**1**) and its semi-synthetic derivatives (**2**,**3**) with highly potent cytotoxicity.

Among the marine alkaloids, renieramycin M (**1**) is a marine bis-1,2,3,4-tetrahydroisoquinolinequinone alkaloid, which was isolated as a major alkaloid from the Thai blue sponge *Xestospongia* sp. found in the Si-Chang Island of Thailand (Figure 1) [14,15]. Interestingly, **1** and its semi-synthetic derivatives, replacing the angelate ester at various new ester side chains at C-22, exhibited a moderate to high potency of cytotoxicity in the nanomolar range against several human cancer cell lines such as colon, lung, and breast carcinomas [16]. In the past several years, the prominent structure–activity relationship studies of **1** and its semi-synthetic derivatives featuring various linear and aromatic ester side chains at C-22 and C-5 have been continuously explored and evaluated for their cytotoxic activity against non-small-cell lung cancer cell lines [17,18], which have been listed as one of the world’s leading causes of death [19,20]. Based on the current findings, the chemically modified ester side chains at C-22 and C-5 displayed a key structure-cytotoxicity relationship. According to their cytotoxic potencies against non-small-cell lung cancer cell lines (H292 and H460), the nitrogen-containing aromatic esters such as the 4-pyridyl ester at C-22 and a small acyl ester such as the acetyl ester at C-5 are essential for the high potencies of **2** and **3**, respectively. However, removing the CN group at C-21 and the ester motif at C-22 causes a drastically diminished cytotoxicity. The additional ester side chain at C-22 and C-5 presumably involves an increase in both hydrophobic interaction and the hydrogen bonding network during the DNA alkylation process to improve the cytotoxicity potency, similar to the mechanism of ecteinascidin 743 (trabectedin), which is an approved tetrahydroisoquinoline-containing antitumor drug. Additionally, the underlying anti-cancer mechanism of **1** and its semi-synthetic derivatives against non-small-cell lung cancer H23, H292, and H460 cell lines was reported to involve the apoptotic and anti-metastatic cell death pathways, which adds to the interest in this series of compounds as potential anti-non-small-cell lung cancer agents [21,22,23,24,25,26].

**Figure 2:**
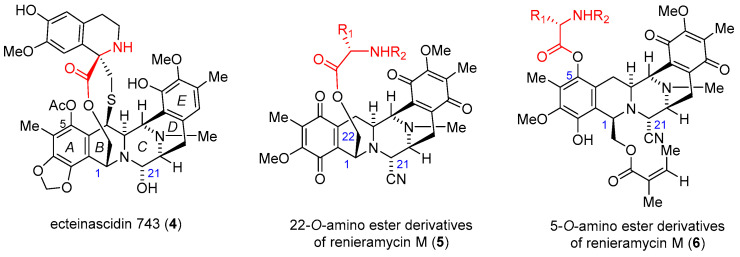
Ecteinascidin 743 as a template of the new series of 22-*O*-amino ester and hydroquinone 5-*O*-amino ester derivatives of **1**.

Based on the structural analysis of ecteinascidin 743 (**4**), the perpendicular ester bridge contains an aminoacyl moiety (red structure) on ring B of the tetrahydroisoquinoline core (Figure 2), which might be available for such interactions with other macromolecules, and two new series of the 22-*O*-amino ester and hydroquinone 5-*O*-amino ester derivatives of **1** (**5** and **6**) have been designed. This work is focused on the semi-synthesis of renieramycin M derivatives bearing an amino ester substituent at either the C-22 or C-5 positions and their cytotoxicity evaluations against the highly metastatic H292 and H460 non-small-cell lung cancer cell lines.

## 2. Results and Discussion

### 2.1. Extraction and Isolation of ***1*** from the Thai Blue Sponge Xestospongia sp.

Compound **1** was isolated from the Thai blue sponge *Xestospongia* sp., collected from the vicinity of Si-Chang Island, the Gulf of Thailand. By using our optimized extraction process for the fresh blue sponge, regarding potassium cyanide pretreatment after neutralization at pH 7 using phosphate buffer solution, **1** was obtained in a high yield [14]. In this work, a sample of the blue sponge *Xestospongia* sp. (ca. 18 kg wet weight equivalent to 3.5 kg dry weight) was collected. Extraction and purification afforded **1** (780 mg) with an isolation yield of 0.02% *w*/*w* relative to the dry sponge. Next, compound **1** was employed as the starting material for the semi-synthesis of the 22-*O*-amino ester and hydroquinone 5-*O*-amino ester derivatives.

### 2.2. Syntheses of 22-O-amino Ester and Hydroquinone 5-O-amino Ester Derivatives of ***1***

**Scheme 1:**
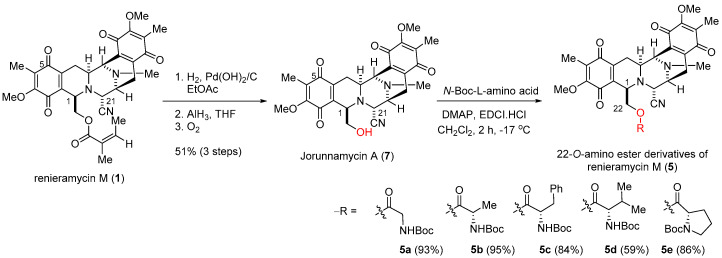
Semi-synthesis of 22-*O*-amino ester derivatives of **1** (**5a**–**5e**).

The preparation of 22-*O*-amino ester derivatives of **1** involved the transformation of **1** to jorunnamycin A (**7**) by the well-known three-step procedure including hydrogenation, hydride reduction, and air oxidation [16,27]. Next, **7** was reacted with the commercially available *N*-*tert*-butyloxycarbonyl (Boc)-protected amino acids including *N*-Boc-l-glycine, *N*-Boc-l-alanine, *N*-Boc-l-phenylalanine, *N*-Boc-l-valine, and *N*-Boc-l-proline under the Steglich esterification in the presence of 1-ethyl-3-(3-dimethylaminopropyl) carbodiimide (EDCI) as a coupling reagent to furnish the desired 22-*O*-amino ester derivatives of **1** in moderate to excellent yields (Scheme 1) [18].

**Scheme 2:**
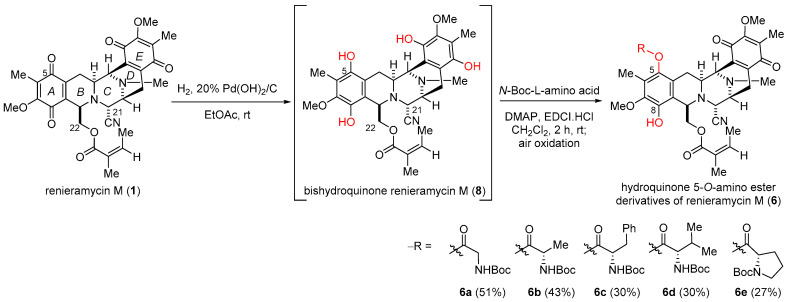
Semi-synthesis of hydroquinone 5-*O*-amino ester derivatives of **1** (**6a**–**6e**).

The series of hydroquinone 5-*O*-amino ester derivatives was prepared by the hydrogenation of **1**, yielding bishydroquinone renieramycin M (**8**), followed by esterification with the selected *N*-Boc-l-amino acids and in situ air oxidation to afford the desired amino ester derivatives (Scheme 2). In more detail, the quinone motifs at rings A and E of **1** were chemoselectively reduced by the Pearlman’s catalyst (Pd(OH)_2_/C) to yield bishydroquinone **8** with its angelate ester at C-22 remaining intact. The Steglich esterification was employed for the regiospecific esterification at the hydroxyl group on C-5, which was located at the least steric hindrance position. After esterification, the hydroquinone at ring E was restored by air oxidation to give back the quinone moiety, yielding the hydroquinone 5-*O* amino ester derivatives (**6**) [15,18]. Although the Steglich esterification with EDCI and *N*, *N*-4-dimethylaminopyridine (DMAP) was selected based on its mild and highly effective condition [28], the hydroquinone 5-*O*-amino ester derivatives were obtained at low to moderate yields due to the steric hindrances surrounding the hydroxyl group at C-5 of **8** and the *N*-Boc-l-amino acid. Compound **1** was commonly recovered from each experiment (7–51%). In addition, the yields of the hydroquinone 5-*O*-amino ester derivatives of **1** were increased when five equivalents of *N*-Boc-l-amino acids were added in one portion. Furthermore, adjustment of the order of addition of **8** and *N*-Boc-l-amino acid was investigated. However, the results were not significantly different. Under these conditions, the major product **6** was formed through mono-esterification of hydroquinol at C-5.

### 2.3. Cytotoxicity of the Semi-Synthetic Amino Ester Derivatives against Non-Small Cell Lung Cancer Cell Lines

The cytotoxicity evaluations against aggressively metastatic H292 and H460 non-small-cell lung cancer cell lines were analyzed by an anti-proliferative assay using the tetrazolium dye, namely 3-(4,5-dimethylthiazol-2-yl)-2,5-diphenyltetrazolium bromide (MTT). As shown in Table 1, the 22-*O*-amino ester **5a**–**5e** and hydroquinone 5-*O*-amino ester **6a**–**6e** exhibited cytotoxicity in the nanomolar range from IC_50_ 3 to 200 nM. Interestingly, the cytotoxicities of the newly synthesized derivatives in the amino ester series were significantly more potent than cisplatin and doxorubicin against the H292 cell line. In case of the H460 cell line, these amino ester derivatives exhibited a cytotoxic activity greater than cisplatin but showed a similar cytotoxicity to doxorubicin. Among the compounds in this series, **5a** displayed the highest cytotoxic activity against the H292 cell line and was more potent than its parent compounds, which are **1** and **7** (7 times higher and 61 times higher), respectively (Table 1, entry 3). Additionally, **6a** exhibited the strongest cytotoxic activity against the H460 cell line with a similar cytotoxicity to **1** and was 27 times more potent than **7** (Table 1, entry **8**). Comparing 22-*O*-amino ester derivatives **5a**–**5e** (Table 1, entries 3–7) and hydroquinone 5-*O*-amino ester derivatives **6a**–**6e** (Table 1, entries 8–12), the series of 22-*O*-amino ester derivatives displayed significant cytotoxic activity against the H292 cell line, while the series of hydroquinone 5-*O*-amino ester derivatives were slightly more cytotoxic against the H460 cell line. Regarding the steric hindrance of the additional ester substituents, bulky groups such as phenylalanine and valine motifs, reduced the cytotoxic potencies, as exemplified by **5c** and **6d**, respectively. It is worth pointing out that the steric hindrance played a larger part in the series of the hydroquinone 5-*O*-amino ester than the series of the 22-*O*-amino ester due to the inherited structural framework of the 1,2,3,4-tetrahydroisoquinolinequinone core.

Considering both semi-synthesis and cytotoxicity results in this work, the 22-*O*-amino ester and hydroquinone 5-*O*-amino ester derivatives were structurally designed based on the addition of the aminoacyl moiety. This motif might play an interesting role in cytotoxicity through its interaction with other macromolecules during DNA alkylation. Moreover, the aminoacyl moiety with a small substituent such as the hydrogen and methyl group of glycine and alanine, respectively, displayed potent cytotoxicity. In contrast, a bulky substituent on the aminoacyl moiety exhibited lower cytotoxicity, as found in the isopropyl group of valine.

Initially, we planned to remove the Boc-protecting group by mild trifluoroacetic acid (TFA) deprotection. However, the preliminary Boc deprotection showed unsuccessful results regarding decomposition of the desired product. Thus, the Boc-protected derivatives were studied further against an antiproliferative MTT assay for cytotoxicity screening. Interestingly, the Boc group featuring a carbamate moiety and a bulky tertiary butyl group may be well suited as a model study to provide more information for future applications of such series of compounds as prodrugs [29,30] or the cytotoxic payload with a linker for antibody conjugation [31], which requires a labile chemical moiety such as carbamate with bulky substitutes representing conjugated molecules. Based on the current investigation, **5a** was obtained in an excellent yield (93%) and exhibited a strong cytotoxicity greater than its parent compounds and the control drugs such as cisplatin and doxorubicin. Thus, **5a** is a potential cytotoxic agent, which can be developed further for non-small cell lung cancer treatment and explored for its anti-cancer mechanism.

## 3. Materials and Methods

### 3.1. General for Synthetic Procedure

Reactions were carried out in oven-dried glassware and magnetically stirred under an argon atmosphere using a calcium chloride U-tube equipped with argon balloon, unless otherwise noted. Room temperature was 25 °C, unless otherwise stated. Commercial solvents and reagents were used as received, unless otherwise explained. Anhydrous solvents were dried over 4Å molecular sieves. Tetrahydrofuran (THF) was distilled immediately prior to use from the reaction of sodium metal and benzophenone. Brine refers to a saturated aqueous solution of sodium chloride. All reactions were monitored by thin-layer chromatography (TLC) performed using aluminum silica gel 60 F254 (Merck). Bands were identified by UV activity. Yields refer to chromatographically and spectroscopically pure compounds unless otherwise stated. Flash column chromatography was performed using 60 Å silica gel (230–400 mesh) in a stationary phase. Optical rotations were measured on a Horiba-SEPA at 25 °C in a 1-decimeter cell (length); concentration (c) is reported in g/mL, unless otherwise stated. Circular dichroism (CD) data were obtained on a JASCO J-720WI. IR spectra were measured on a Hitachi 260-10 spectrophotometer. The ^1^H- and ^13^C-NMR spectra were recorded at 400 MHz on a JEOL-JNM AL 400 FT-NMR spectrometer (supplementary files). The mass spectra were recorded on a JEOL-JMS 700 instrument with a direct inlet system operating at 70 eV. Accurate mass spectra were obtained with an Agilent 6540 UHD Q-TOF LC/MS spectrometer using fast atom bombardment (FAB^+^) ionization and a Zorbax Eclipse Plus C18 HPLC column (3.0 × 100 mm). ^1^H-NMR chemical shifts are reported as δ values in ppm relative to tetramethylsilane (TMS, 0.00 ppm) or residual CHCl_3_ (7.27 ppm). ^1^H-NMR coupling constants (*J*) are reported in Hertz (Hz). Unless otherwise indicated, deuterochloroform (CDCl_3_) served as an internal standard (77.0 ppm) for all ^13^C spectra. The numbering of carbons in natural products and derivatives was adopted from initial isolation reports.

### 3.2. Isolation of Renieramycin M (***1***)

#### 3.2.1. Sample Collection

The sponge *Xestospongia* sp. was collected by scuba diving in the vicinity of Si-Chang Island at a depth of 3–5 m with assistance from the Aquatic Recourses Research Institute, Chulalongkorn University and permission from the Department of Fisheries, Ministry of Agriculture and Cooperatives, Thailand (0510.2/8234, Date 28 October 2019). The fresh sponge was previously characterized based on its color and outer skeleton, including its light bluish-gray color, bulbous surface lobes, the numerous and moderate sizes of its oscules, and its easily crumbled texture [14]. The sample was kept frozen at −20 °C until use.

#### 3.2.2. Extraction and Isolation

The standard methods for isolation and purification of the blue sponge *Xestospongia* sp. involved potassium cyanide pre-treatment followed by methanolic extraction [14]. The collected marine organism, around 18 kg, was homogenized and suspended in phosphate buffer solution (pH 7). Then, a solution of 10% potassium cyanide in phosphate buffer solution (pH 7) was added dropwise to the suspension until it reached the concentration of potassium cyanide at 10 mM. During potassium cyanide pre-treatment step, the suspension was gradually checked and maintained at pH 7 to avoid the formation of the toxic hydrogen cyanide gas. The mixture was continuously stirred for 5 h. Thereafter, the mixture was macerated for 48 h with methanol. The extract was filtered, and the filtrate was concentrated under reduced pressure to obtain the aqueous methanolic solution. The maceration was repeated for 4 cycles. The combined aqueous methanolic solution was partitioned with hexane and ethyl acetate consecutively. In each partition, the relative volume of each organic solvent compared to the aqueous methanolic solution was 1:1 ratio. The obtained aqueous layers were discarded as the hazardous waste for further proper cyanide waste disposal [32]. Then, the volatile solvents were removed to give the crude residues. The ethyl acetate crude residue was subjected to silica gel chromatography using the gradient solvent mixture of hexane, ethyl acetate, and methanol. Finally, the purified products were combined and recrystallized in a 1:1 mixture of hexane and dichloromethane to obtain **1** (780 mg).

### 3.3. Semi-Synthesis Procedures

#### 3.3.1. Preparation of Jorunnamycin A (**7**)

Compound **7** was semi-synthesized from **1** based on the reported protocol [27]. Compound **1** (100.0 mg, 0.1739 mmol) was weighed into the reaction vessel and dissolved in ethyl acetate (EtOAc) (30 mL). Then, to the resulting orange solution, 20% Pd(OH)_2_ on carbon (50.0 mg, 50% *w*/*w*), was added. A hydrogen balloon was attached to the reaction flask. The heterogeneous reaction was stirred vigorously at room temperature (25 °C) under 1 atm for 5 h. The reaction mixture was filtered through a pad of celite and washed with EtOAc (20 mL × 3) and CHCl_3_ (20 mL × 3). The filtrates were combined and concentrated in vacuo to yield bishydroquinone renieramycin M as a colorless solid. The product was employed in the next step without further purification. A solution of bishydroquinone renieramycin M (83.1 mg, 0.143 mmol) in dry THF (10.0 mL) was cooled at −17 °C and stirred in an argon atmosphere. A freshly prepared solution of AlH_3_ (0.5 M, 2.3 mL, 1.147 mmol) was added dropwise over a 10 min period. The reaction mixture was stirred at −17 °C for 4 h. The reaction was quenched by the addition of water (2 mL) and CHCl_3_ (20 mL) at −17 °C. The mixture was slowly warmed up to room temperature. After 20 min, Na_2_SO_4_ (10 g) was added and an oxygen balloon was attached to the reaction flask. The reaction mixture was stirred vigorously at room temperature overnight (10 h). After air oxidation, the reaction mixture was filtered and washed with CHCl_3_ (50 mL × 3). The filtrates were combined and concentrated in vacuo until 20 mL of the crude mixture remained. The crude product was washed with 5% Na_2_CO_3_ aqueous solution. The organic layer was separated by a separating funnel and the aqueous layer was extracted with CHCl_3_ (20 mL × 3). The organic layers were combined, washed with brine (50 mL), dried over anhydrous Na_2_SO_4_, filtered, and concentrated in vacuo. Purification by silica gel flash chromatography and eluting with n-hexane:EtOAc (1:1 to 1:9) gave 43.8 mg (51%) of **7** as an orange amorphous solid, along with 25.6 mg (26%) of recovered **1** and 5.8 mg (7%) of decyanoyl jorunnamycin A. Jorunnamycin A (**7**); ^1^H-NMR (CDCl_3_, 400 MHz) δ 4.17 (1H, d, *J* = 2.1 Hz, 21-H), 4.08 (1H, d, *J* = 2.0 Hz, 11-H), 4.03 (3H, s, 17-OCH_3_), 3.99 (3H, s, 7-OCH_3_), 3.89 (1H, br d, *J* = 2.1 Hz, 1-H), 3.71 (1H, dd, *J* = 8.7, 2.1 Hz, 22-H_a_), 3.50 (1H, br d, *J* = 8.7 Hz 22-H_b_), 3.42 (1H, br d, *J* = 5.0 Hz, 13-H), 3.17 (1H, dt, *J* = 8.5, 2.0 Hz, 3-H), 2.93 (1H, dd, *J* = 13.0, 2.0 Hz, 4-H_α_), 2.83 (1H, dd, *J* = 16.0, 5.0 Hz, 14-H_α_), 2.30 (3H, s, NCH_3_), 2.27 (1H, d, *J* = 16.0 Hz, 14-H_β_), 1.94 (6H, s, 6-CH_3_ and 16-CH_3_), 1.41 (1H, ddd, *J* = 13.0, 8.5, 1.8 Hz, 4-H_β_); ^13^C-NMR (CDCl_3_, 100 MHz) δ 186.3 (C-15), 185.5 (C-5), 182.3 (C-18), 181.4 (C-8), 155.5 (C-7), 155.4 (C-17), 141.7 (C-20), 141.4 (C-10), 136.1 (C-9), 135.6 (C-19), 128.9 (C-6), 128.6 (C-16), 116.9 (21-CN), 64.3 (C-22), 61.1 (17-OCH_3_), 61.1 (7-OCH_3_), 59.1 (C-21), 58.0 (C-1), 54.5 (C-13), 54.3 (C-3), 54.2 (C-11), 41.6 (NCH_3_), 25.4 (C-4), 21.5 (C-14), 8.8 (6-CH_3_), 8.7 (16-CH_3_).

#### 3.3.2. General Procedure for Preparation of 22-*O*-amino Ester Derivatives of Renieramycin M (**5a**–**5e**)

Compound **7** (10.0 mg, 0.02 mmol, 1 equivalent) was weighed in an oven dried-rounded bottom flask and dissolved in dry CH_2_Cl_2_ (2 mL). The resulting orange solution was cooled at −17 °C and 1-ethyl-3-(3-dimethylaminopropyl) carbodiimide (EDCI.HCl, 4.0 mg, 0.02 mmol, 1 equivalent) and *N*, *N*-4-dimethylaminopyridine (DMAP, 3.0 mg, 0.02 mmol, 1 equivalent) were added, followed by *N*-Boc-l-amino acid (15–25 mg, 0.10 mmol, 5 equivalent). The reaction mixture was stirred at −17 °C for 2 h in an argon atmosphere. The reaction mixture was quenched by the addition of water (5 mL). The organic layer was separated by a separating funnel and the aqueous layer was extracted with CHCl_3_ (10 mL × 3). The organic layers were combined, washed with brine (30 mL), dried over anhydrous Na_2_SO_4_, filtered, and concentrated in vacuo. Purification by silica gel flash chromatography and eluting with n-hexane:EtOAc (4:1 to 2:1) gave 22-*O*-amino ester derivatives of **1**.

22-*O*-(*N*-Boc-l-glycine) ester of renieramycin M (**5a**): 11.6 mg (93%); yellow amorphous solid. ${\left[\alpha \right]}_{D}^{25}$ +161.7 (*c*: 0.0035, CHCl_3_); CD Δε (c: 9 μM, methanol, 20 °C) +0.02 (371), −0.3 (314), −0.2 (315), −1.0 (300), −2.4 (283), −1.3 (269), −0.3 (257), −0.9 (242), −1.3 (227); IR (KBr) 3414, 2978, 2943, 2855, 1751, 1717, 1655, 1616, 1508, 1452, 1369, 1310, 1281, 1236, 1161 cm^−1^; ^1^H-NMR (CDCl_3_, 400 MHz) δ 4.84 (1H, br s, 3′-NH), 4.47 (1H, dd, *J* = 10.8, 2.4 Hz, 22-H_a_), 4.06 (1H, br d, *J* = 2.4 Hz, 21-H), 4.03 (3H, s, 7-OCH_3_), 4.03 (1H, overlapped, 11-H), 4.01 (3H, s, 17-OCH_3_), 4.01 (1H, overlapped, 1-H), 3.95 (1H, overlapped, 22-H_b_), 3.78 (1H, dd, *J* = 18.6, 6.0 Hz, 2′-H_a_), 3.49 (1H, dd, *J* = 18.6, 6.0 Hz, 2′-H_b_), 3.37 (1H, br d, *J* = 7.4 Hz, 13-H), 3.10 (1H, dt, *J* = 12.0, 2.6 Hz, 3-H), 2.94 (1H, dd, *J* = 17.3, 2.6 Hz, 4-H_α_), 2.73 (1H, dd, *J* = 20.7, 7.4 Hz, 14-H_α_), 2.33 (1H, d, *J* = 20.7 Hz, 14-H_β_), 2.30 (3H, s, NCH_3_), 1.96 (3H, s, 6-CH_3_), 1.95 (3H, s, 16-CH_3_), 1.42 (9H, s, 3 × 6′-CH_3_), 1.35 (1H, overlapped, 4-H_β_); ^13^C-NMR (CDCl_3_, 100 MHz) δ 186.6 (C-15), 185.2 (C-5), 182.4 (C-18), 181.1 (C-8), 169.8 (C-1′), 155.5 (C-4′), 155.4 (C-7), 155.3 (C-17), 142.1 (C-10), 142.0 (C-20), 135.1 (C-9), 135.0 (C-19), 128.9 (C-6), 128.3 (C-16), 116.9 (21-CN), 80.2 (C-5′), 64.1 (C-22), 61.1 (17-OCH_3_), 61.1 (7-OCH_3_), 59.0 (C-21), 55.7 (C-1), 54.6 (C-3), 54.2 (C-13), 54.1 (C-11), 42.3 (C-2′), 41.5 (NCH_3_), 28.3 (3 × 6′-CH_3_), 25.2 (C-4), 21.2 (C-14), 8.9 (6-CH_3_), 8.6 (16-CH_3_). FAB^+^ HRMS *m*/*z* 651.6931 ([M + H]^+^, calculated for C_33_H_39_N_4_O_10_, 651.6929).

22-*O*-(*N*-Boc-l-alanine) ester of renieramycin M (**5b**): 12.3 mg (95%); yellow amorphous solid. ${\left[\alpha \right]}_{D}^{25}$ −75.8 (*c*: 0.0024, CHCl_3_); CD Δε (c: 9 μM, methanol, 20 °C) −4.8 (355), −3.5 (333), −2.1 (313), −2.8 (299), −9.3 (289), −16.1 (280), −5.0 (270), +8.9 (257), +5.4 (244), +0.2 (234); IR (KBr) 3393, 2978, 2940, 2855, 1765, 1717, 1653, 1616, 1506, 1456, 1369, 1306, 1234, 1155, 1058 cm^−1^; ^1^H-NMR (CDCl_3_, 400 MHz) δ 4.69 (1H, d, *J* = 7.5 Hz, 3′-NH), 4.45 (1H, br d, *J* = 11.6 Hz, 22-H_a_), 4.08 (1H, br d, *J* = 11.6 Hz, 22-H_b_), 4.01 (1H, d, *J* = 2.4 Hz, 21-H), 3.96 (3H, s, 7-OCH_3_), 3.96 (1H, overlapped, 11-H), 3.94 (3H, s, 17-OCH_3_), 3.92 (1H, overlapped, 1-H), 3.89 (1H, overlapped, 2′-H), 3.33 (1H, br d, *J* = 7.4 Hz, 13-H), 3.02 (1H, dt, *J* = 11.6, 3.2 Hz, 3-H), 2.82 (1H, br d, *J* = 16.8, Hz, 4-H_α_), 2.67 (1H, dd, *J* = 20.9, 7.4 Hz, 14-H_α_), 2.30 (1H, d, *J* = 20.9 Hz, 14-H_β_), 2.22 (3H, s, NCH_3_), 1.87 (3H, s, 6-CH_3_), 1.37 (1H, overlapped, 4-H_β_), 1.87 (3H, s, 16-CH_3_), 1.37 (1H, overlapped, 4-H_β_), 1.31 (9H, s, 3 × 6′-CH_3_), 1.03 (3H, d, *J* = 7.6 Hz, 7′-CH_3_); ^13^C-NMR (CDCl_3_, 100 MHz) δ 186.5 (C-15), 185.2 (C-5), 182.4 (C-18), 181.1 (C-8), 172.6 (C-1′), 155.6 (C-4′), 155.4 (C-7), 154.7 (C-17), 141.9 (C-10), 141.9 (C-20), 135.1 (C-9), 134.8 (C-19), 128.7 (C-6), 128.1 (C-16), 116.8 (21-CN), 79.9 (C-5′), 63.1 (C-22), 61.1 (17-OCH_3_), 61.0 (7-OCH_3_), 58.4 (C-21), 56.5 (C-1), 54.5 (C-3), 54.3 (C-13), 54.1 (C-11), 48.7 (C-2′), 41.5 (NCH_3_), 28.2 (3 × 6′-CH_3_), 25.2 (C-4), 21.0 (C-14), 18.3 (7′-CH_3_), 8.8 (6-CH_3_), 8.5 (16-CH_3_). FAB^+^ HRMS *m*/*z* 665.2824 ([M + H]^+^, calculated for C_34_H_41_N_4_O_10_, 665.2823).

22-*O*-(*N*-Boc-l-phenylalanine) ester of renieramycin M (**5c**): 12.4 mg (84%); yellow amorphous solid. ${\left[\alpha \right]}_{D}^{25}$ +29.7 (*c*: 0.0017, CHCl_3_); CD Δε (c: 9 μM, methanol, 20 °C) −6.7 (356), −4.4 (327), −4.0 (300), −9.5 (292), −23.1 (281), −3.5 (269), +11.4 (256), +5.4 (244), −2.8 (234); IR (KBr) 3387, 2963, 2928, 2855, 1719 (br), 1655, 1612, 1560, 1508, 1499, 1458, 1368, 1306, 1261, 1234, 1159, 1107, 1084, 1049, 1030, 957, 801, 700 cm^−1^; ^1^H-NMR (CDCl_3_, 400 MHz) δ 7.29 (3H, m 2 × 10′-H & 11′-H), 7.05 (2H, br d, *J* = 6.3 Hz, 2 × 9′-H), 4.71 (1H, d, *J* = 8.1 Hz, 3′-H), 4.30 (2H, dd, *J* = 11.4, 3.0 Hz, 22-H_2_), 4.24 (1H, d, *J* = 7.8 Hz, 2′-H), 4.11 (1H, overlapped, 21-H), 4.08 (1H, overlapped, 1-H), 4.00 (6H, s, 17 and 7-OCH_3_), 3.87 (1H, br, 11-H), 3.34 (1H, br d, *J* = 7.1 Hz, 13-H), 3.07 (1H, dt, *J* = 11.4, 3.8 Hz, 3-H), 2.91 (1H, br d, *J* = 16.8, Hz, 4-H_α_), 2.84 (2H, d, *J* = 5.7 Hz, 7′-H ), 2.45 (1H, dd, *J* = 21.0, 7.1 Hz, 14-H_α_), 2.30 (1H, d, *J* = 21.0 Hz, 14-H_β_), 2.28 (3H, s, NCH_3_), 1.92 (3H, s, 6-CH_3_), 1.86 (3H, s, 16-CH_3_), 1.38 (1H, overlapped, 4-H_β_), 1.32 (9H, s, 3 × 6′-CH_3_); ^13^C-NMR (CDCl_3_, 100 MHz) δ 186.4 (C-15), 185.1 (C-5), 182.4 (C-18), 181.1 (C-8), 171.7 (C-1′), 155.5 (C-4′), 155.3 (C-7), 154.7 (C-17), 142.2 (C-10), 141.9 (C-20), 135.7 (C-9), 134.9 (C-19), 129.3 (C-8′), 129.1 (C-9′), 128.7 (C-10′), 128.6 (C-6), 128.0 (C-16), 127.2 (C-11′), 116.8 (21-CN), 80.0 (C-5′), 64.3 (C-22), 62.0 (C-2′), 61.1 (17-OCH_3_), 61.0 (7-OCH_3_), 58.6 (C-21), 55.9 (C-1), 54.6 (C-3), 54.3 (C-13), 54.2 (C-11), 41.4 (NCH_3_), 38.3 (C-7′), 28.1 (3 × 6′-CH_3_), 25.0 (C-4), 21.4 (C-14), 8.8 (6-CH_3_), 8.6 (16-CH_3_). FAB^+^ HRMS *m*/*z* 741. 3134 ([M + H]^+^, calculated for C_40_H_45_N_4_O_10_, 741.3136).

22-*O*-(*N*-Boc-l-valine) ester of renieramycin M (**5d**): 8.1 mg (59%); yellow amorphous solid. ${\left[\alpha \right]}_{D}^{25}$ +135.4 (*c*: 0.0023, CHCl_3_); CD Δε (c: 6 μM, methanol, 20 °C) −1.2 (353), −0.8 (326), −2.0 (301), −5.1 (289), −8.1 (281), −2.9 (270), +2.3 (257), −0.2 (240), −1.2 (224); IR (KBr) 3404, 2969, 2940, 1741, 1717, 1655, 1616, 1506, 1456, 1371, 1310, 1236, 1157 cm^−1^; ^1^H-NMR (CDCl_3_, 400 MHz) δ 4.73 (1H, br d, *J* = 9.6 Hz, 3′-H), 4.29 (2H, d, *J* = 2.7 Hz, 22-H_2_), 4.08 (1H, d, *J* = 2.0 Hz, 21-H), 4.07 (1H, overlapped, 2′-H), 4.06 (3H, s, 7-OCH_3_), 4.04 (1H, overlapped, 1-H), 4.03 (1H, overlapped, 11-H), 4.00 (3H, s, 17-OCH_3_), 3.40 (1H, dt, *J* = 7.8, 2.0 Hz, 13-H), 3.08 (1H, dt, *J* = 11.4, 2.4 Hz, 3-H), 2.90 (1H, dd, *J* = 16.8, 2.4 Hz, 4-Hα), 2.77 (1H, dd, *J* = 21.0, 7.8 Hz, 14-H_α_), 2.30 (1H, d, *J* = 21.0 Hz, 14-H_β_), 2.28 (3H, s, NCH_3_), 1.95 (3H, s, 6-CH_3_), 1.93 (3H, s, 16-CH_3_), 1.43 (1H, d, *J* = 3.0 Hz, 4-H_β_), 1.36 (9H, s, 3 × 6′-CH_3_), 1.26 (1H, m, 7′-H), 0.80 & 0.73 (each 3H, d, *J* = 6.9 Hz, 2 × 8′-CH_3_); ^13^C-NMR (CDCl_3_, 100 MHz) δ 186.5 (C-15), 185.1 (C-5), 182.4 (C-18), 181.1 (C-8), 171.8 (C-1′), 155.6 (C-4′), 155.5 (C-7), 155.2 (C-17), 141.9 (C-10), 141.8 (C-20), 135.1 (C-9), 134.8 (C-19), 128.5 (C-6), 127.8 (C-16), 116.7 (21-CN), 79.8 (C-5′), 63.7 (C-22), 61.1 (17-OCH_3_), 61.1 (7-OCH_3_), 58.6 (C-2′), 58.4 (C-21), 56.4 (C-1), 54.6 (C-3), 54.5 (C-13), 54.1 (C-11), 41.5 (NCH_3_), 30.8 (C-7′), 28.2 (3 × 6′-CH_3_), 25.1 (C-4), 21.2 (C-14), 18.9 & 17.5 (8′-CH_3_), 8.8 (6-CH_3_), 8.7 (16-CH_3_). FAB^+^ HRMS *m*/*z* 693.3143 ([M + H]^+^, calculated for C_36_H_45_N_4_O_10_, 693.3136).

22-*O*-(*N*-Boc-l-proline) ester of renieramycin M (**5e**): 11.6 mg (86%); yellow amorphous solid. ${\left[\alpha \right]}_{D}^{25}$ +161.1 (*c*: 0.0044, CHCl_3_); CD Δε (c: 14 μM, methanol, 20 °C) +1.6 (417), −1.3 (389), −3.8 (358), −2.6 (328), −2.5 (300), −12.5 (282), −3.1 (270), +6.8 (258), +2.8 (243), −1.1 (222), +2.5 (212), +7.3 (207); IR (KBr) 2974, 2955, 2940, 1749, 1695, 1655, 1616, 1450, 1400, 1373, 1312, 1236, 1188, 1159, 1084 cm^−1^; ^1^H-NMR (CDCl_3_, 400 MHz) δ 4.35 (1H, dd, *J* = 11.6, 2.0 Hz, 22-H_a_), 4.29 (1H, dd, *J* = 11.6, 2.4 Hz, 22-H_b_), 4.27 (1H, m, 2′-H), 4.10 (1H, br, 21-H), 4.01 (1H, overlapped, 11-H), 4.02 (3H, s, 7-OCH_3_), 3.99 (3H, s, 17-OCH_3_), 3.99 (1H, overlapped, 1-H), 3.40 (1H, m, 13-H), 3.30 (2H, m, 9′-H_2_), 3.09 (1H, br d, *J* = 11.6 Hz, 3-H), 2.95 (1H, br d, *J* = 17.2 Hz, 4-H_α_), 2.76 (1H, dd, *J* = 20.7, 7.6 Hz, 14-H_α_), 2.33 (1H, d, *J* = 20.7 Hz, 14-H_β_), 2.28 (3H, s, NCH_3_), 1.93 (3H, s, 6-CH_3_), 1.91 (3H, s, 16-CH_3_), 1.75 (2H, m, 8′-H_2_), 1.34 (9H, s, 3 × 6′-CH_3_), 1.43 (1H, overlapped, 4-H_α_), 1.26 (2H, m, 7′-H_2_); ^13^C-NMR (CDCl_3_, 100 MHz) δ 186.4 (C-15), 185.3 (C-5), 182.4 (C-18), 181.1 (C-8), 172.5 (C-1′), 155.7 (C-4′), 155.5 (C-7), 154.0 (C-17), 142.0 (C-10), 141.8 (C-20), 135.3 (C-9), 134.7 (C-19), 128.4 (C-6), 127.8 (C-16), 117.0 (21-CN), 79.7 (C-5′), 65.2 (C-22), 63.7 (C-2′), 61.0 (17-OCH_3_ & 7-OCH_3_), 58.5 (C-21), 58.6 (C-4), 56.4 (C-1), 54.6 (C-3), 54.3 (C-11), 54.2 (C-13), 46.5 (C-9′), 41.5 (NCH_3_), 31.6 (C-7′), 29.8, (C-8′), 28.3 (3 × 6′-CH_3_), 25.2 (C-4), 21.2 (C-14), 8.8 (6-CH_3_), 8.6 (16-CH_3_). FAB^+^ HRMS *m*/*z* 691.2970 ([M + H]^+^, calculated for C_36_H_43_N_4_O_10_, 691.2980).

#### 3.3.3. General Procedure for Preparation of Hydroquinone 5-*O*-amino Ester Derivatives of Renieramycin M (**6a**–**6e**)

Compound **1** (37.0 mg, 0.065 mmol) was dissolved in EtOAc (20 mL) and 20% Pd(OH)_2_ on carbon (19.0 mg, 50% *w*/*w*) was added. A hydrogen balloon was attached to the reaction flask. The heterogeneous reaction was stirred vigorously at room temperature (25 °C) for 5 h. The reaction was filtered through a pad of celite and washed with EtOAc (20 mL × 3) and CHCl_3_ (20 mL × 3). The filtrates were combined and concentrated in vacuo to yield bishydroquinone renieramycin M (**8**) as a colorless solid (quant). The product was employed in the next step without further purification. A solution of **8** (10.0 mg, 0.017 mmol, 1 equivalent) in dry CH_2_Cl_2_ (2 mL) was stirred at room temperature (25 °C) and EDCI.HCl (3.3 mg, 0.17 mmol, 1 equivalent) and DMAP (2.1 mg, 0.17 mmol, 1 equivalent) were added, followed by *N*-Boc-l-amino acid (10–25 mg, 0.085 mmol, 5 equivalent). The reaction mixture was stirred for 2 h in an argon atmosphere. The reaction mixture was quenched by the addition of water (5 mL). The organic layer was separated by a separating funnel and the aqueous layer was extracted with CHCl_3_ (10 mL × 3). The organic layers were combined, washed with brine (30 mL), dried over anhydrous Na_2_SO_4_, filtered, and concentrated in vacuo. Purification by silica gel flash chromatography and eluting with n-hexane:EtOAc (3:1) gave hydroquinone 5-*O*-amino ester derivatives of **1**.

5-*O*-(*N*-Boc-l-glycine) ester derivatives of renieramycin M (**6a**): 4.8 mg (51%); yellow amorphous solid. ${\left[\alpha \right]}_{D}^{25}$ +161.7 (*c*: 0.0007, CHCl_3_); CD Δε (c: 11 μM, methanol, 20 °C) +1.8 (420), 0.0 (384), −1.0 (353), −0.4 (323), 0.0 (298), +1.9 (282), +3.1 (264), +2.0 (244), +1.5 (229), +2.6 (217); IR (KBr) 3393, 2961, 2926, 2855, 1775, 1717, 1653, 1616, 1462, 1369, 1261, 1234, 1153, 1094, 1059, 1030, 802 cm^−1^; ^1^H-NMR (CDCl_3_, 400 MHz) δ 5.93 (1H, qq, *J* = 7.4, 1.6 Hz, 26-H), 5.70 (1H, s, 8-OH), 5.00 (1H, br s, 3′-NH), 4.44 (1H, br d, *J* = 11.0 Hz, 22-H_a_), 4.24 (2H, d, *J* = 4.4 Hz, 2′-H_2_), 4.16 (1H, br d, *J* = 5.0 Hz, 1-H), 4.04 (1H, d, *J* = 2.8 Hz, 21-H), 3.98 (1H, dd, *J* = 11.0, 5.0 Hz, 22-H_b_), 3.90 (3H, s, 17-OCH_3_), 3.88 (1H, br d, *J* = 2.2 Hz, 11-H), 3.71 (3H, s, 7-OCH_3_), 3.29 (1H, br d, *J* = 8.0 Hz, 13-H), 3.12 (1H, dt, *J* = 12.2, 2.2 Hz, 3-H), 2.68 (1H, dd, *J* = 21.4, 8.0 Hz, 14-H_α_), 2.40 (1H, dd, *J* = 15.2, 2.2 Hz, 4-H_α_), 2.22 (1H, d, *J* = 21.4 Hz, 14-H_β_), 2.20 (3H, s, NCH_3_), 2.02 (3H, s, 6-CH_3_), 1.86 (3H, s, 16-CH_3_), 1.80 (3H, d, *J* = 7.4 Hz, 27-H_3_), 1.61 (3H, br s, 28-H_3_), 1.55 (1H, overlapped, 4-H_β_), 1.42 (9H, s, 3 × 6′-CH_3_); ^13^C-NMR (CDCl_3_, 100 MHz) δ 186.1 (C-15), 182.8 (C-18), 180.9 (C-1′), 167.1 (C-24), 155.4 (C-4′), 155.4 (C-17), 143.8 (C-7), 143.3 (C-8), 141.9 (C-20), 140.1 (C-26), 138.7 (C-5), 135.4 (C-19), 129.7 (C-16), 126.8 (C-25), 124.1 (C-10), 122.5 (C-6), 117.5 (21-CN), 117.2 (C-9), 80.2 (C-5′), 64.3 (C-22), 61.2 (17-OCH_3_), 60.8 (7-OCH_3_), 59.4 (C-21), 56.5 (C-1), 55.2 (C-3), 54.8 (C-13), 54.8 (C-11), 42.2 (C-2′), 41.5 (NCH_3_), 28.3 (3 × 6′-CH_3_), 27.9 (C-4), 21.1 (C-14), 20.6 (28-CH_3_), 15.8 (27-CH_3_), 10.0 (16-CH_3_), 8.7 (6-CH_3_). FAB^+^ HRMS *m*/*z* 735.3250 ([M + H]^+^, calculated for C_38_H_47_N_4_O_11_, 735.3242).

5-*O*-(*N*-Boc-l-alanine) ester of renieramycin M (**6b**): 7.8 mg (43%); yellow amorphous solid. ${\left[\alpha \right]}_{D}^{25}$ +34.1 (*c*: 0.0039, CHCl_3_); CD Δε (c: 11 μM, methanol, 20 °C) +1.7 (401), −0.4 (381), −1.7 (357), −0.5 (331), +0.8 (307), +1.9 (294), +5.2 (282), +7.0 (267), +5.4 (250), +2.4 (236); IR (KBr) 3393, 2976, 2938, 1765, 1717, 1653, 1616, 1458, 1369, 1304, 1234, 1153, 1059, 1059 cm^−1^; ^1^H-NMR (CDCl_3_, 400 MHz) δ 6.01 (1H, qq, *J* = 7.4, 1.6 Hz, 26-H), 5.78 (1H, s, 8-OH), 5.00 (1H, br s, 3′-NH), 4.59 (1H, m, 2′-H), 4.48 (1H, dd, *J* = 11.2, 2.8 Hz, 22-H_a_), 4.31 (1H, dd, *J* = 5.4, 2.8 Hz, 1-H), 4.11 (1H, d, *J* = 2.4 Hz, 21-H), 4.02 (1H, dd, *J* = 11.2, 5.4 Hz, 22-H_b_), 3.97 (3H, s, 17-OCH_3_), 3.94 (1H, br d, *J* = 2.6 Hz, 11-H), 3.78 (3H, s, 7-OCH_3_), 3.35 (1H, br d, *J* = 7.4 Hz, 13-H), 3.20 (1H, dt, *J* = 12.2, 2.6 Hz, 3-H), 2.75 (1H, dd, *J* = 21.1, 7.4 Hz, 14-H_α_), 2.54 (1H, br dd, *J* = 15.2, 2.6 Hz, 4-H_α_), 2.28 (1H, d, *J* = 21.1 Hz, 14-H_β_), 2.27 (3H, s, NCH_3_), 2.11 (3H, s, 6-CH_3_), 1.92 (3H, s, 16-CH_3_), 1.88 (3H, dq, *J* = 7.4, 1.6 Hz, 27-H_3_), 1.72 (3H, d, *J* = 7.2 Hz, 7′-H_3_), 1.71 (3H, br s, 28-H_3_), 1.64 (1H, dd, *J* = 15.2, 12.2 Hz, 4-H_β_), 1.47 (9H, s, 3 × 6′-CH_3_); ^13^C-NMR (CDCl_3_, 100 MHz) δ 186.1 (C-15), 182.8 (C-18), 171.6 (C-1′), 167.1 (C-24), 155.2 (C-17), 155.0 (C-4′), 143.8 (C-7), 143.3 (C-8), 142.0 (C-20), 140.0 (C-26), 138.7 (C-5), 135.4 (C-19), 129.0 (C-16), 126.8 (C-25), 124.1 (C-10), 122.7 (C-6), 117.5 (21-CN), 117.1 (C-9), 80.1 (C-5′), 64.8 (C-22), 61.2 (17-OCH_3_), 60.8 (7-OCH_3_), 59.7 (C-21), 56.4 (C-1), 55.4 (C-3), 54.8 (C-13), 54.8 (C-11), 49.3 (C-2′), 41.5 (NCH_3_), 28.3 (3 × 6′-CH_3_), 27.5 (C-4), 21.2 (C-14), 20.6 (28-CH_3_), 18.7 (7′-CH_3_), 15.8 (27-CH_3_), 10.1 (16-CH_3_), 8.7 (6-CH_3_). FAB^+^ HRMS *m*/*z* 749.3392 ([M + H]^+^, calculated for C_39_H_49_N_4_O_11_, 749.3398).

5-*O*-(*N*-Boc-l-phenylalanine) ester of renieramycin M (**6c**): 5.5 mg (30%); yellow amorphous solid. ${\left[\alpha \right]}_{D}^{25}$ −28.1 (*c*: 0.0031, CHCl_3_); CD Δε (c: 5 μM, methanol, 20 °C) −6.1 (358), −3.4 (330), +0.2 (299), +3.9 (288), +15.6 (271), +11.7 (254), +3.5 (235), +18.6 (227), +30.7 (219), +24.9 (209); IR (KBr) 3393, 2934, 1763, 1717, 1653, 1616, 1499, 1456, 1368, 1303, 1234, 1161, 1078, 1059, 770, 700 cm^−1^; ^1^H-NMR (CDCl_3_, 400 MHz) δ 7.38 (3H, m, 2 × 10′-H & 11′-H), 7.33 (2H, dt, *J* = 8.8, 2.8 Hz, 2 × 9′-H), 6.01 (1H, qq, *J* = 7.4, 1.6 Hz, 26-H), 5.78 (1H, s, 8-OH), 4.87 (1H, br d, *J* = 8.0 Hz, 3′-NH), 4.81 (1H, br, *J* = 10.0, 4.0 Hz, 2′-H), 4.48 (1H, dd, *J* = 10.8, 2.0 Hz, 22-H_a_), 4.32 (1H, t, *J* = 5.0 Hz, 1-H), 4.10 (1H, d, *J* = 2.0 Hz, 21-H), 4.03 (1H, dd, *J* = 10.8, 5.0 Hz, 22-H_b_), 3.94 (1H, d, *J* = 2.4 Hz, 11-H), 3.90 (3H, s, 17-OCH_3_), 3.78 (3H, s, 7-OCH_3_), 3.56 (1H, dd, *J* = 13.8, 4.4 Hz, 7′-H_a_), 3.35 (1H, br d, *J* = 7.6 Hz, 13-H), 3.20 (1H, dt, *J* = 12.0, 2.4 Hz, 3-H), 3.08 (1H, dd, *J* = 13.8, 10.2 Hz, 7′-H_b_), 2.74 (1H, dd, *J* = 20.7, 7.6 Hz, 14-H_α_), 2.62 (1H, br d, *J* = 13.6 Hz, 4-H_α_), 2.29 (1H, d, *J* = 20.7 Hz, 14-H_β_), 2.26 (3H, s, NCH_3_), 2.09 (3H, s, 6-CH_3_), 1.92 (3H, s, 16-CH_3_), 1.88 (3H, dq, *J* = 7.6, 1.6 Hz, 27-H_3_), 1.71 (3H, d, *J* = 1.6 Hz, 28-H_3_), 1.67 (1H, dd, *J* = 13.6, 12.0 Hz, 4-H_β_), 1.38 (9H, s, 3 × 6′-CH_3_); ^13^C-NMR (CDCl_3_, 100 MHz) δ 186.1 (C-15), 182.8 (C-18), 170.1 (C-1′), 167.1 (C-24), 155.2 (C-4′), 155.2 (C-17), 143.8 (C-7), 143.3 (C-8), 142.0 (C-20), 140.0 (C-26), 138.8 (C-5), 136.3 (C-19), 135.4 (C-8′), 129.3 (2 × C-10′), 128.7 (C-16), 128.7 (2 × C-9′), 127.1 (C-11′), 126.8 (C-25), 124.1 (C-10), 123.1 (C-6), 117.6 (21-CN), 117.6 (C-9), 80.1 (C-5′), 64.9 (C-22), 61.2 (17-OCH_3_), 60.8 (7-OCH_3_), 59.7 (C-2′), 59.7 (C-21), 56.4 (C-1), 55.5 (C-3), 54.9 (C-13), 54.9 (C-11), 41.5 (NCH_3_), 38.3 (C-7′), 28.2 (3 × 6′-CH_3_), 27.1 (C-4), 21.2 (C-14), 20.6 (28-CH_3_), 15.9 (27-CH_3_), 10.1 (16-CH_3_), 8.7 (6-CH_3_). FAB^+^ HRMS *m*/*z* 825.3715 ([M + H]^+^, calculated for C_45_H_53_N_4_O_11_, 825.3712).

5-*O*-(*N*-Boc-l-valine derivative of renieramycin M (**6d**): 4.0 mg (30%); yellow amorphous solid. ${\left[\alpha \right]}_{D}^{25}$ −11.5 (*c*: 0.0026, CHCl_3_); CD Δε (c: 10 μM, methanol, 20 °C) −1.8 (400), −1.8 (386), −1.7 (369), −1.3 (348), −0.5 (325), −0.4 (298), −0.6 (288), −1.8 (273), −4.0 (251), −3.5 (240), −3.0 (229); IR (KBr) 3393, 2972, 2940, 1761, 1717, 1653, 1616, 1462, 1369, 1306, 1234, 1153 cm^−1^; ^1^H-NMR (CDCl_3_, 400 MHz) δ 6.01 (1H, qq, *J* = 7.6, 1.6 Hz, 26-H), 5.76 (1H, s, 8-OH), 4.97 (1H, d, *J* = 9.2 Hz, 3′-NH), 4.51 (1H, dd, *J* = 9.6, 4.8 Hz, 2′-H), 4.39 (1H, dd, *J* = 9.6, 3.0 Hz, 22-H_a_), 4.33 (1H, dd, *J* = 5.6, 3.0 Hz, 1-H), 4.07 (1H, d, *J* = 2.4 Hz, 21-H), 3.99 (1H, overlapped, *J* = 4.8 Hz, 22-H_b_), 3.96 (3H, s, 17-OCH_3_), 3.95 (1H, overlapped, 11-H), 3.77 (3H, s, 7-OCH_3_), 3.34 (1H, br d, *J* = 7.8 Hz, 13-H), 3.20 (1H, dt, *J* = 12.4, 2.0 Hz, 3-H), 2.74 (1H, dd, *J* = 20.7, 7.8 Hz, 14-H_α_), 2.66 (1H, d, *J* = 16.0 Hz, 4-H_α_), 2.45 (1H, m, 7′-H), 2.31 (1H, overlapped, 14-H_β_), 2.26 (3H, s, NCH_3_), 2.08 (3H, s, 6-CH_3_), 1.92 (3H, s, 16-CH_3_), 1.85 (3H, dq, *J* = 7.6, 1.3 Hz, 27-H_3_), 1.71 (3H, br s, 28-H_3_), 1.67 (1H, d, *J* = 16.0 Hz, 4-H_β_), 1.47 (9H, s, 3 × 6′-CH_3_), 1.19 & 1.08 (each 3H, d, *J* = 5.2 Hz, 2 × 8′-H3); ^13^C-NMR (CDCl_3_, 100 MHz) δ 186.2 (C-15), 182.5 (C-18), 178.1 (C-1′), 167.1 (C-24), 155.8 (C-4′), 155.2 (C-17), 143.9 (C-7), 143.3 (C-8), 142.0 (C-20), 140.0 (C-26), 138.9 (C-5), 135.4 (C-19), 128.9 (C-16), 126.8 (C-25), 124.3 (C-10), 123.1 (C-6), 117.5 (21-CN), 117.2 (C-9), 80.1 (C-5′), 65.4 (C-22), 61.2 (17-OCH_3_), 60.9 (7-OCH_3_), 60.1 (C-2′), 58.8 (C-21), 56.3 (C-1), 55.9 (C-3), 54.9 (C-13), 54.9 (C-11), 41.5 (NCH_3_), 30.7 (C-7′), 28.3 (3 × 6′-CH_3_), 27.3 (C-4), 21.4 (C-14), 20.6 (28-CH_3_), 19.9 (C-8′), 15.8 (27-CH_3_), 10.3 (16-CH_3_), 8.7 (6-CH_3_). FAB^+^ HRMS *m*/*z* 777.3705 ([M + H]^+^, calculated for C_41_H_53_N_4_O_11_, 777.3712).

5-*O*-acyl-*N*-Boc-l-glycine derivative of renieramycin M (**6e**): 4.1 mg (27%); yellow amorphous solid. ${\left[\alpha \right]}_{D}^{25}$ +92.0 (*c*: 0.0020, CHCl_3_); CD Δε (c: 8 μM, methanol, 20 °C) 0.0 (299), +0.1 (279), +0.3 (263), +0.2 (246), +0.3 (232), +0.5 (222); IR (KBr) 2978, 2938, 1717, 1655, 1616, 1506, 1456, 1369, 1308, 1261, 1236, 1161, 1109, 1094, 1061, 955, 802 cm^−1^; ^1^H-NMR (CDCl_3_, 400 MHz) δ 5.99 (1H, qq, *J* = 7.5, 1.2 Hz, 26-H), 4.63 (2H, m, 22-H_2_), 4.59 (1H, overlapped, 2′-H), 4.28 (1H, m, 1-H), 4.13 (1H, d, *J* = 2.4 Hz, 21-H), 3.95 (3H, s, 17-OCH_3_), 3.90 (1H, overlapped, 11-H), 3.71 (3H, s, 7-OCH_3_), 3.55 (1H, br d, *J* = 7.6 Hz, 13-H), 3.45 (2H, m, 9′-H_2_), 3.19 (1H, br d, *J* = 11.6, 3-H), 2.73 (1H, dd, *J* = 21.0, 7.6 Hz, 14-H_α_), 2.52 (1H, br d, *J* = 14.4 Hz, 4-H_α_), 2.32 (1H, d, *J* = 21.0 Hz, 14-H_β_), 2.25 (3H, s, NCH_3_), 2.17 (2H, overlapped, 8′-H), 2.09 (3H, s, 6-CH_3_), 1.93 (2H, t, *J* = 10 Hz, 7′-H_2_), 1.91 (3H, s, 16-CH_3_), 1.87 (3H, dq, *J* = 7.5, 1.8 Hz, 27-H_3_), 1.69 (3H, br s, 28-H_3_), 1.61 (2H, m, 9′-H), 1.41 (1H, overlapped, 4-H_β_), 1.41 (9H, s, 6′-CH_3_); ^13^C-NMR (CDCl_3_, 100 MHz) δ 186.0 (C-15), 182.9 (C-18), 171.0 (C-1′), 166.6 (C-24), 155.1 (C-4′), 154.4 (C-17), 144.1 (C-7), 148.8 (C-8), 142.2 (C-20), 139.7 (C-26), 138.6 (C-5), 135.2 (C-19), 129.8 (C-16), 126.8 (C-25), 124.7 (C-10), 124.5 (C-6), 117.3 (21-CN), 116.9 (C-9), 80.3 (C-5′), 79.9 (C-22), 61.1 (17-OCH_3_), 60.8 (7-OCH_3_), 60.0 (C-2′), 58.6 (C-21), 56.5 (C-9′), 56.3 (C-1), 55.7 (C-3), 55.5 (C-13), 54.8 (C-11), 41.5 (NCH_3_), 31.4 (C-7′), 31.1 (C-8′), 29.9 (C-4), 28.4 (3 × 6′-CH_3_), 21.3 (C-14), 20.6 (28-CH_3_), 15.8 (27-CH_3_), 10.5 (16-CH_3_), 8.7 (6-CH_3_). FAB^+^ HRMS *m*/*z* 775.3550 ([M + H]^+^, calculated for C_41_H_51_N_4_O_11_, 775.3555).

#### 3.3.4. Cytotoxic Evaluations against Non-Small-Cell Lung Cancer Cell Lines

The new series of 22-*O*-amino esters, **5a**–**5e**, and hydroquinone 5-*O*-amino esters, **6a**–**6e**, were evaluated for their cytotoxicity against metastatic non-small cell lung carcinoma lines H292 and H460 according to the 3-(4,5-dimethylthiazol-2-yl)-2,5-diphenyltetrazolium bromide (MTT) assay [33]. Cisplatin and doxorubicin were used as the positive controls. A solution of 0.2% dimethylsulfoxide (DMSO) was used as the negative control. The samples were solubilized with 100% DMSO to 10 μM and diluted to 0.5 μM with DMSO. Then, the resulting sample solutions were diluted with Roswell Park Memorial Institute (RPMI) medium to prepare the serial dilution. Non-small-cell lung cancer cells were seeded into a 96-well plate at a density of 2 × 10^3^ cells/well and allow to adhere overnight. After that, cells were treated with various concentrations of each derivative that was dissolved in RPMI medium containing no more than 0.2% DMSO for 72 h. Then, cells were incubated with 0.5 mg/mL MTT for 4 h. The absorbance of the formazan products solubilized by DMSO was measured at 570 nm. The percentage of cell viability was calculated with respect to non-treated control cells. The mean IC_50_ values ± standard deviations (SD) were obtained from three independent experiments. Each experiment was performed in triplicate in at least five concentrations of the tested compounds on three different days. The IC_50_ value of each experiment was determined and averaged using GraphPad Prism software.

## 4. Conclusions

In summary, a new series of 22-*O*-amino ester and hydroquinone 5-*O*-amino ester derivatives of **1** was successfully prepared, staring from natural bis-1,2,3,4-tetrahydroisoquinolinequinone marine alkaloid (**1**) as the precursor. The chemical modification of 22-*O*-amino ester derivatives involved a reduction in the angelate ester (23–51% yield) and esterification sequence (59–93% yield), while the chemical modification of hydroquinone 5-*O*-amino ester derivatives involved hydrogenation and esterification (27–51% yield of two steps). All synthesized compounds were tested for their cytotoxicity against the metastatic non-small-cell lung cancer H292 and H460 cell lines. The series of 22-*O*-amino ester derivatives were found to be more cytotoxic than that of hydroquinone 5-*O*-amino ester derivatives. In addition, 22-*O*-amino ester derivatives showed a more promising cytotoxic activity against the H292 cell line than that of the H460 cell line. Among the compounds in these series, the most potent derivative is the 22-*O*-(*N*-Boc-l-glycine) ester of renieramycin M (**5a**, IC_50_ 3.56 nM), which was 7-fold more toxic than **1** (IC_50_ 24.56 nM) and 61-fold more toxic than **7** (IC_50_ 217.43 nM) against H292 cells. This new derivative will be further studied for its anti-lung cancer mechanism of action and its potentiality as a cytotoxic agent for the treatment of non-small-cell lung cancer.

## Figures and Tables

**Table 1:** Cytotoxicity of 22-*O*-amino ester and hydroquinone 5-*O*-amino ester derivatives of **1**.

Entry	Compound	O–R	H292	H460
			IC_50_ ± S.D. (nM)	IC_50_ ± S.D. (nM)
1	1	Angeloyl	24.56 ± 1.12	6.50 ± 0.39
2	7	H	217.43 ± 21.67	164.30 ± 11.07
3	5a	*N*-Boc-l-Gly	3.56 ± 0.62	9.94 ± 0.82
4	5b	*N*-Boc-l-Ala	10.32 ± 1.95	19.75 ± 2.38
5	5c	*N*-Boc-l-Phe	110.50 ± 2.53	51.43 ± 5.67
6	5d	*N*-Boc-l-Val	32.39 ± 3.33	20.95 ± 3.53
7	5e	*N*-Boc-l-Pro	66.55 ± 9.47	45.91 ± 6.94
8	6a	*N*-Boc-l-Gly	10.24 ± 0.94	6.14 ± 0.68
9	6b	*N*-Boc-l-Ala	24.03 ± 2.61	15.94 ± 1.32
10	6c	*N*-Boc-l-Phe	57.35 ± 5.36	42.72 ± 4.91
11	6d	*N*-Boc-l-Val	127.20 ± 2.65	200.53 ± 29.27
12	6e	*N*-Boc-l-Pro	85.00 ± 8.62	43.28 ± 0.68
13	cisplatin	-	12.13 × 10^3^ ± 1.12 × 10^3^	8.15 × 10^3^ ± 0.64 × 10^3^
14	doxorubicin	-	350.70 ± 32.94	33.70 ± 4.56

H292 and H460: human non-small cell lung cancer cell lines.

## Supplementary Materials

The following are available online at https://www.mdpi.com/1660-3397/18/8/418/s1, Figures S1–S20: ^1^H and ^13^C-NMR spectra of **5a**–**5e** and **6a**–**6e**.
