# Supplementary material for: Chemistry of Renieramycins. Part 19: Semi-Syntheses of 22-O-Amino Ester and Hydroquinone 5-O-Amino Ester Derivatives of Renieramycin M and Their Cytotoxicity against Non-Small-Cell Lung Cancer Cell Lines
Source: Mar Drugs. 2020 Aug 10;18(8):418. doi: 10.3390/md18080418 (PMC7460379; doi:10.3390/md18080418)
Supplement: Supplementary file 1 [file marinedrugs-18-00418-s001.pdf]

Supporting Information

Chemistry of Renieramycins. Part 19: Semi-syntheses of 22-*O*-amino ester and hydroquinone 5-*O*-amino ester derivatives of renieramycin M and their cytotoxicity against non-small-cell lung cancer cell lines

Supakarn Chamni [1,2,*], Natchanun Sirimangkalakitti [3], Pithi Chanvorachote [4], Khanit Suwanborirux [2], Naoki Saito [3,*]

1 Natural Products and Nanoparticles Research Unit (NP[2]), Chulalongkorn University, Bangkok, Thailand; supakarn.c@pharm.chula.ac.th
2 Department of Pharmacognosy and Pharmaceutical Botany, Faculty of Pharmaceutical Sciences, Chulalongkorn University, Pathumwan, Bangkok 10330, Thailand; supakarn.c@pharm.chula.ac.th
3 Graduate School of Pharmaceutical Sciences, Meiji Pharmaceutical University, 2-522-1 Noshio, Kiyose,Tokyo 204-8588, Japan; naoki@my-pharm.ac.jp
4 Department of Pharmacology and Physiology, Faculty of Pharmaceutical Sciences, Chulalongkorn University, Pathumwan, Bangkok 10330, Thailand; pithi_chan@yahoo.com
* Correspondence: supakarn.c@pharm.chula.ac.th; Tel.: +66-218-8357 (S.C.) and naoki@my-pharm.ac.jp; Tel.: +81-424-95-8792 (N.Sa.)

Table of Figures **Page**

Figure S1. ^{1}H NMR (400 MHz) spectrum of **5a** in $CDCl_3$ S3

Figure S2. ^{13}C NMR (100 MHz) spectrum of **5a** in $CDCl_3$ S3

Figure S3. ^{1}H NMR (400 MHz) spectrum of **5b** in $CDCl_3$ S4

Figure S4. ^{13}C NMR (100 MHz) spectrum of **5b** in $CDCl_3$ S4

Figure S5. ^{1}H NMR (400 MHz) spectrum of **5c** in $CDCl_3$ S5

Figure S6. ^{13}C NMR (100 MHz) spectrum of **5c** in $CDCl_3$ S5

Figure S7. ^{1}H NMR (400 MHz) spectrum of **5d** in $CDCl_3$ S6

Figure S8. ^{13}C NMR (100 MHz) spectrum of **5d** in $CDCl_3$ S6

Figure S9. ^{1}H NMR (400 MHz) spectrum of **5e** in $CDCl_3$ S7

Figure S19. ^{13}C NMR (100 MHz) spectrum of **5e** in $CDCl_3$ S7

Figure S11. ^{1}H NMR (400 MHz) spectrum of **6a** in $CDCl_3$ S8

Figure S12. ^{13}C NMR (100 MHz) spectrum of **6a** in $CDCl_3$ S8

Figure S13. ^{1}H NMR (400 MHz) spectrum of **6b** in $CDCl_3$ S9

Figure S14. ^{13}C NMR (100 MHz) spectrum of **6b** in $CDCl_3$ S9

Figure S15. ^{1}H NMR (400 MHz) spectrum of **6c** in $CDCl_3$ S10

Figure S16. ^{13}C NMR (100 MHz) spectrum of **6c** in $CDCl_3$ S10

Figure S17. ^{1}H NMR (400 MHz) spectrum of **6d** in $CDCl_3$ S11

Figure S18. ^{13}C NMR (100 MHz) spectrum of **6d** in $CDCl_3$ S11

Figure S19. ^{1}H NMR (400 MHz) spectrum of **6e** in $CDCl_3$ S12

Figure S20. ^{13}C NMR (100 MHz) spectrum of **6e** in $CDCl_3$ S12

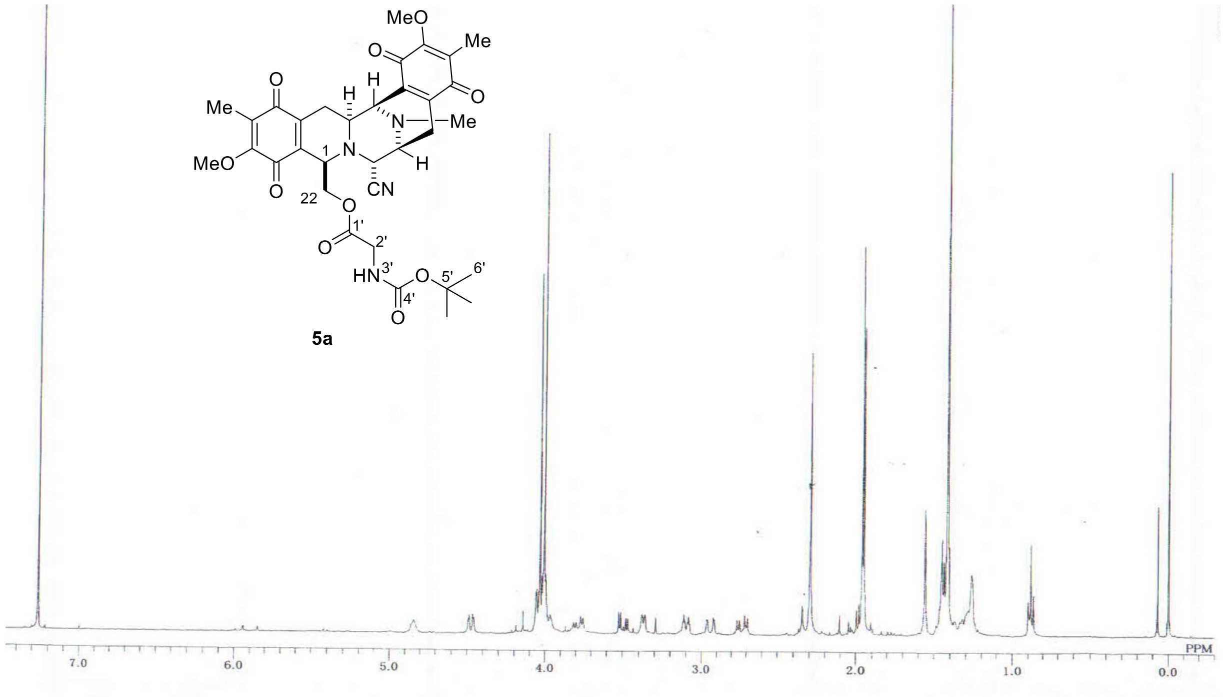

Figure S1. ^{1}H NMR (400 MHz) spectrum of **5a** in $CDCl_3$

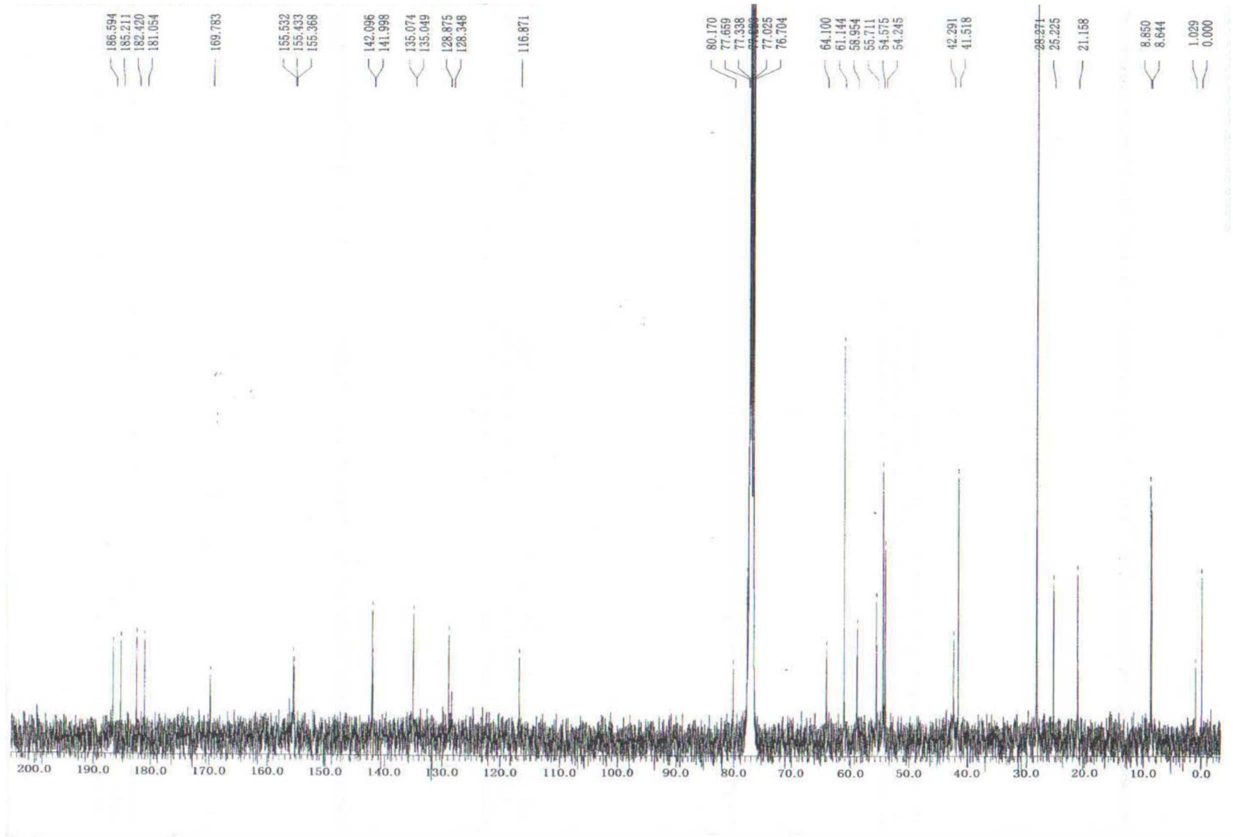

Figure S2. ^{13}C NMR (100 MHz) spectrum of **5a** in $CDCl_3$

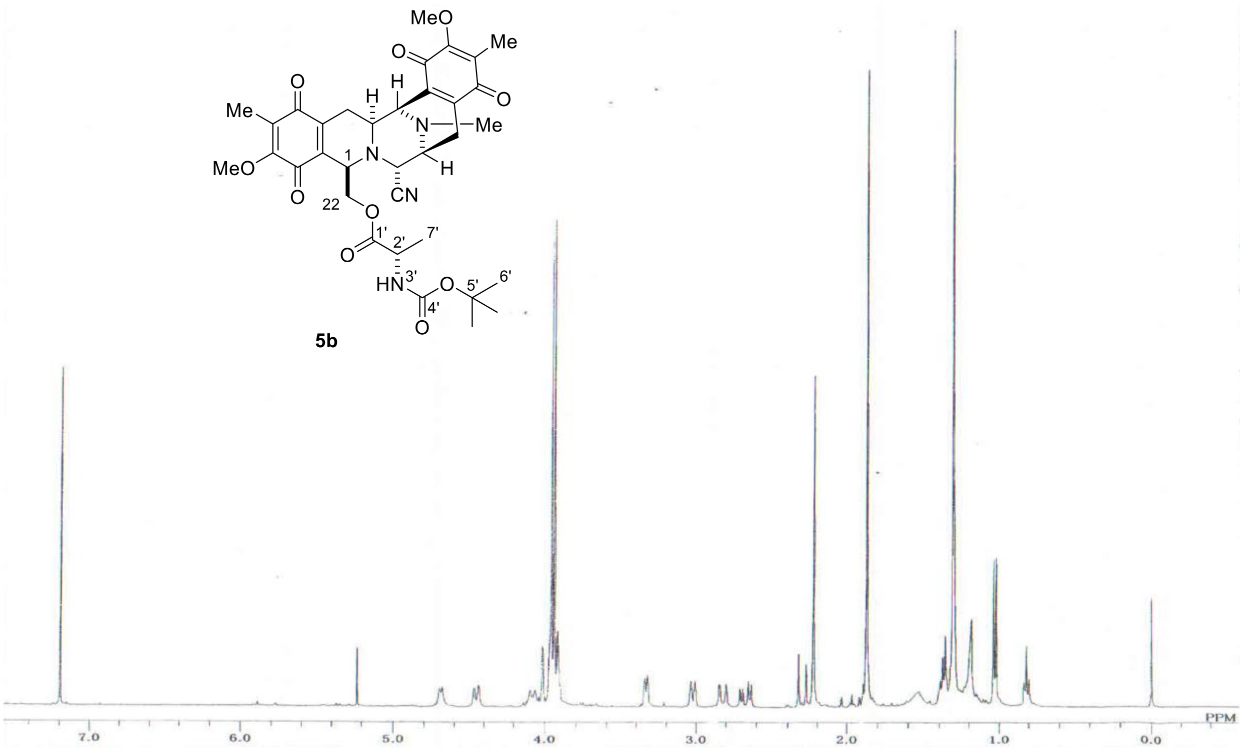

Figure S3. ^{1}H NMR (400 MHz) spectrum of **5b** in $CDCl_3$

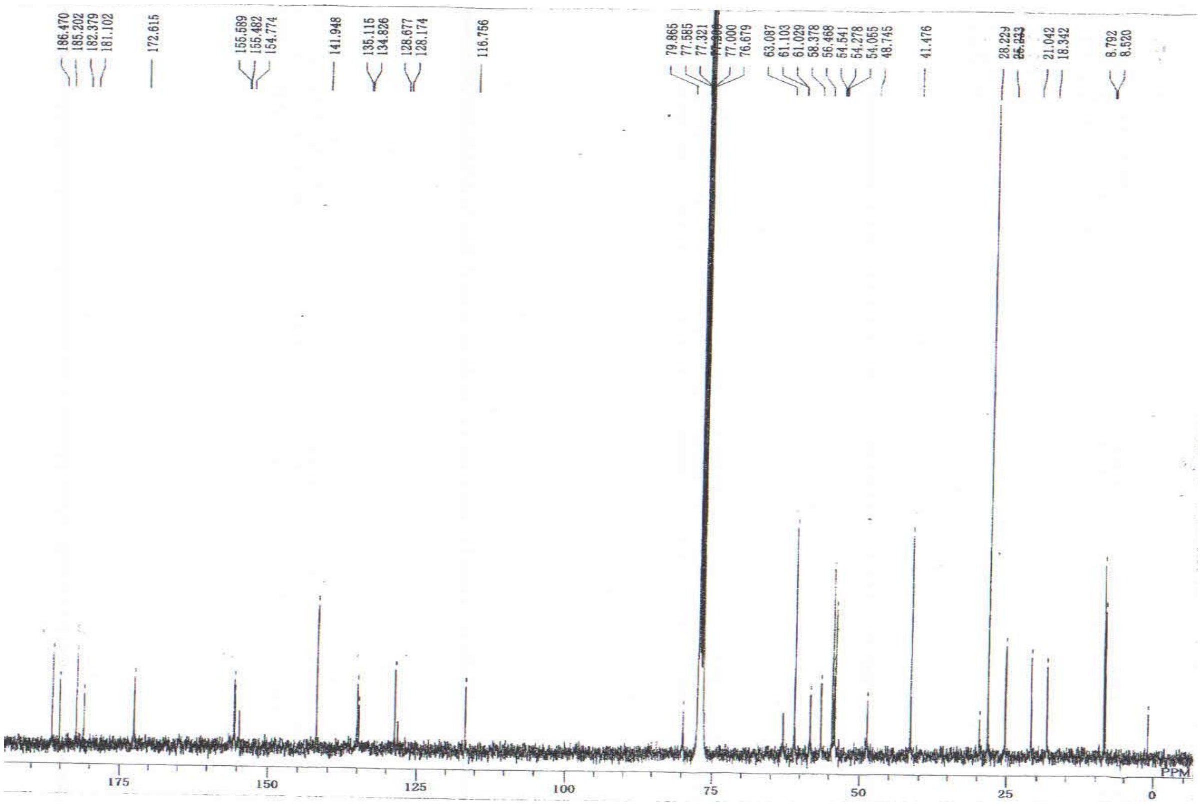

Figure S4. ^{13}C NMR (100 MHz) spectrum of **5b** in $CDCl_3$

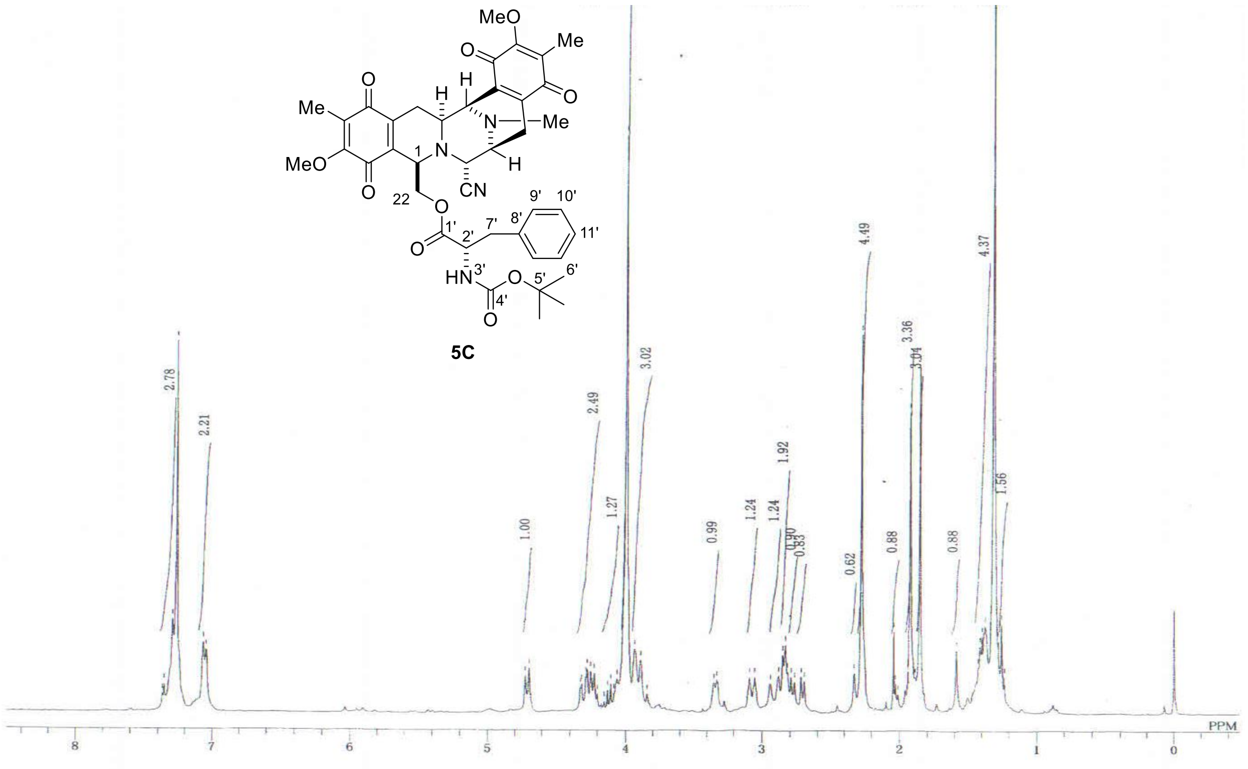

Figure S5. ^{1}H NMR (400 MHz) spectrum of **5c** in $CDCl_3$

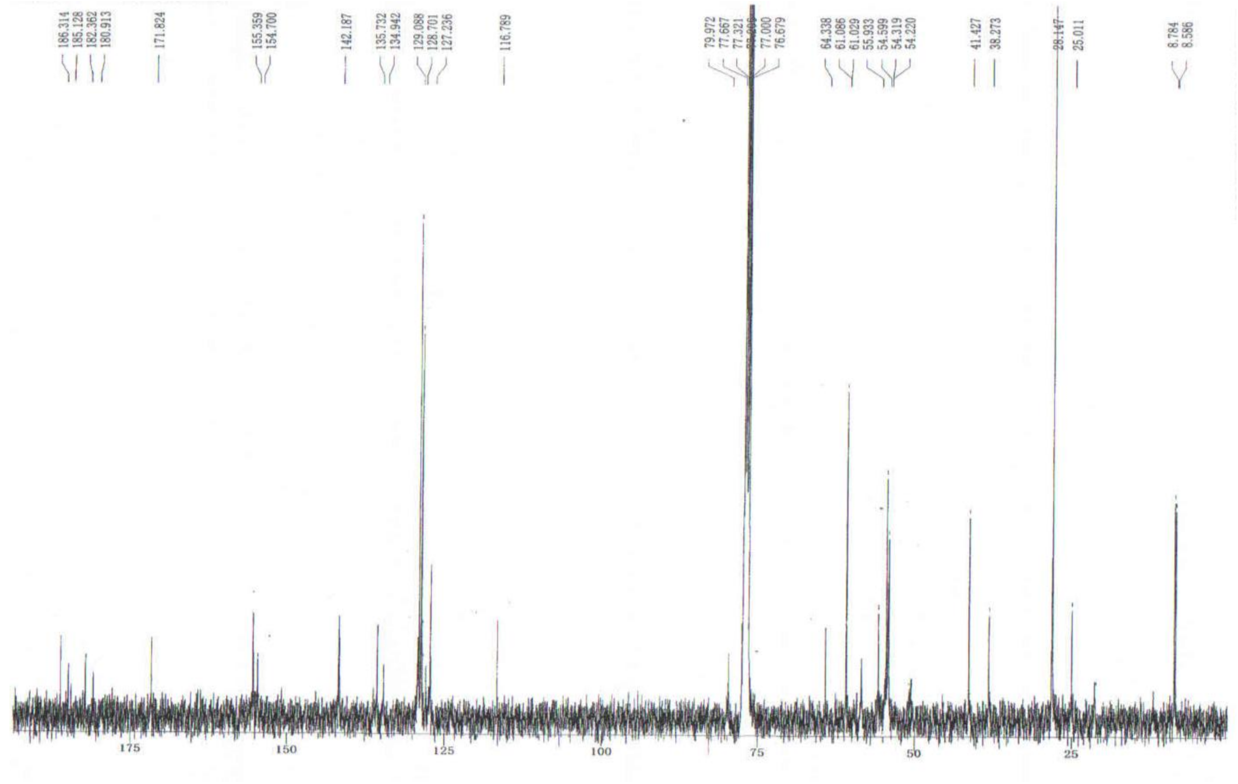

Figure S6. ^{13}C NMR (100 MHz) spectrum of **5c** in $CDCl_3$

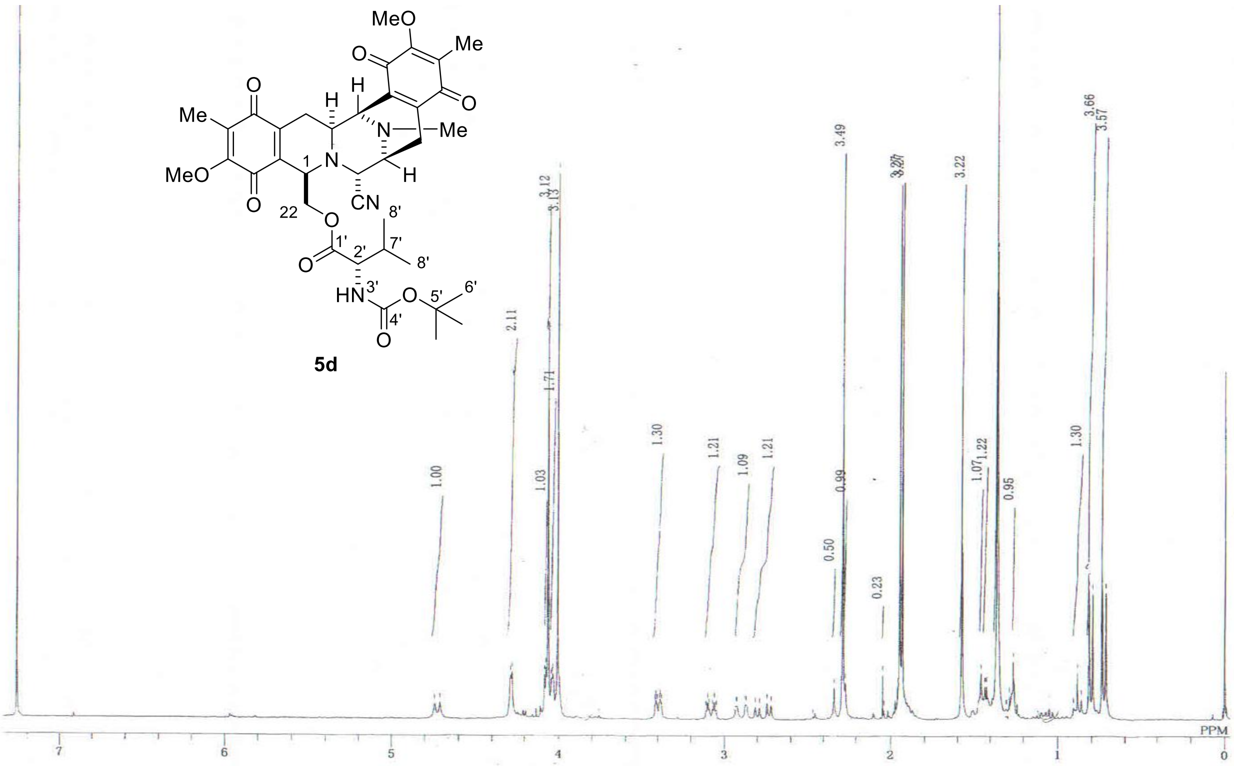

Figure S7. ^{1}H NMR (400 MHz) spectrum of **5d** in $CDCl_3$

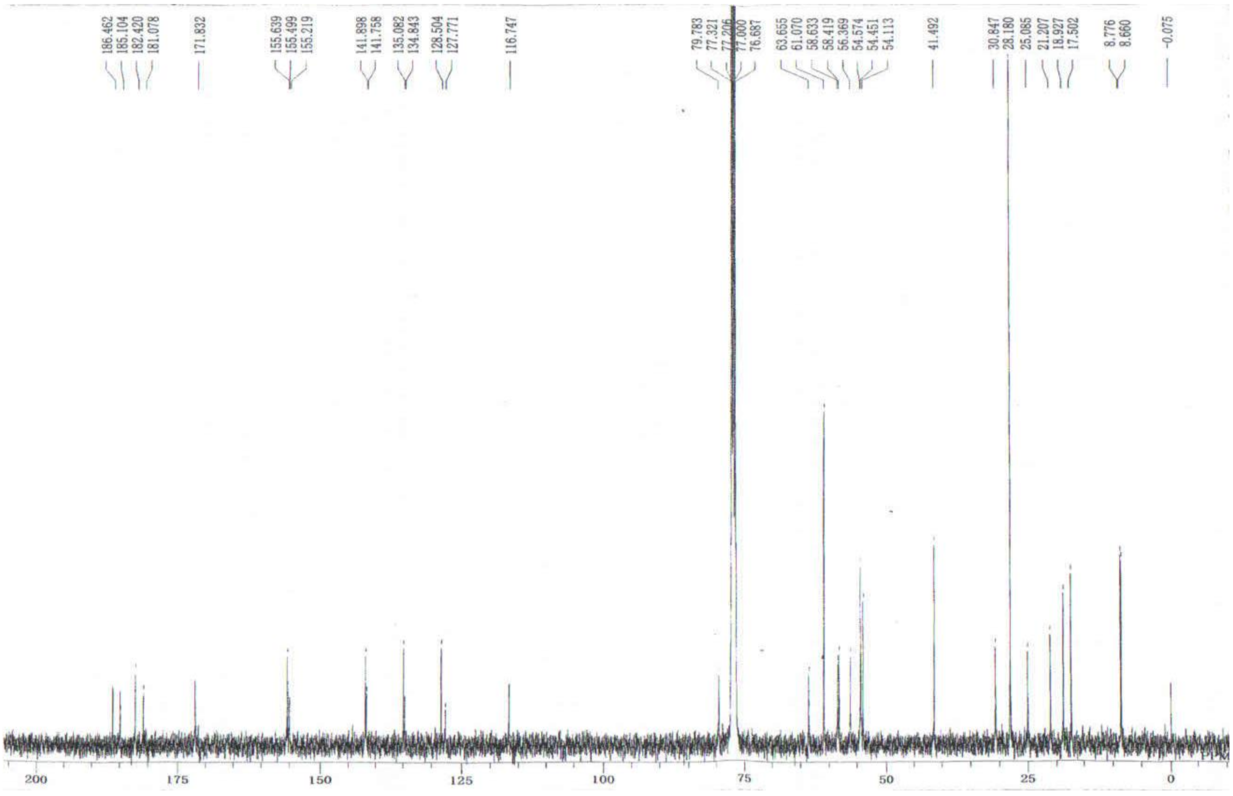

Figure S8. ^{13}C NMR (100 MHz) spectrum of **5d** in $CDCl_3$

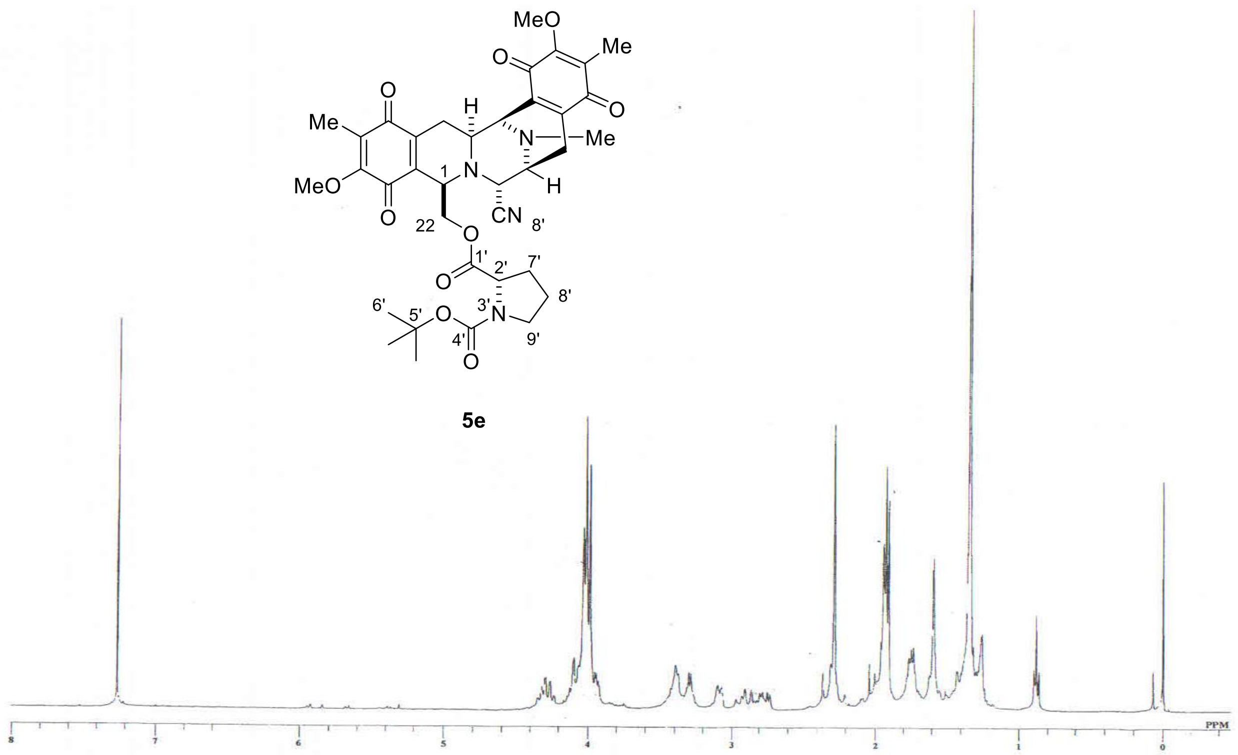

Figure S9. ^{1}H NMR (400 MHz) spectrum of **5e** in $CDCl_3$

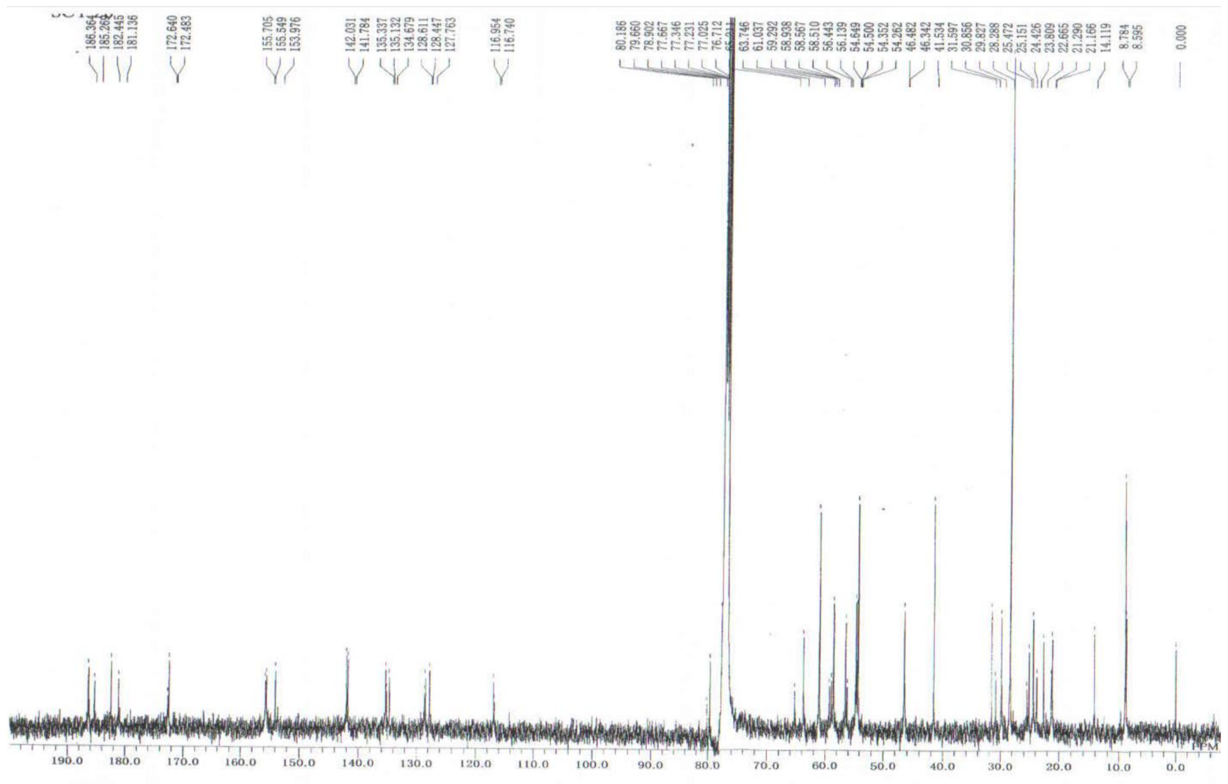

Figure S19. ^{13}C NMR (100 MHz) spectrum of **5e** in $CDCl_3$

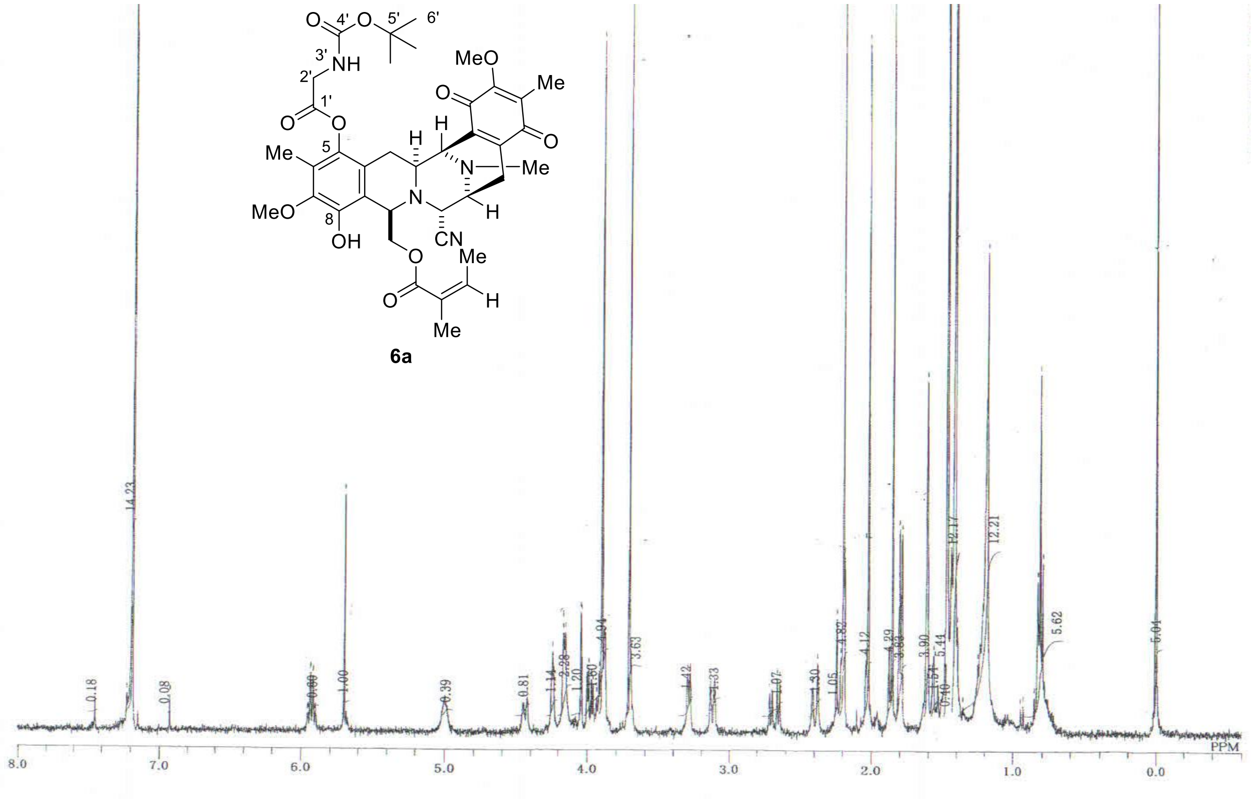

Figure S11. ^{1}H NMR (400 MHz) spectrum of **6a** in $CDCl_3$

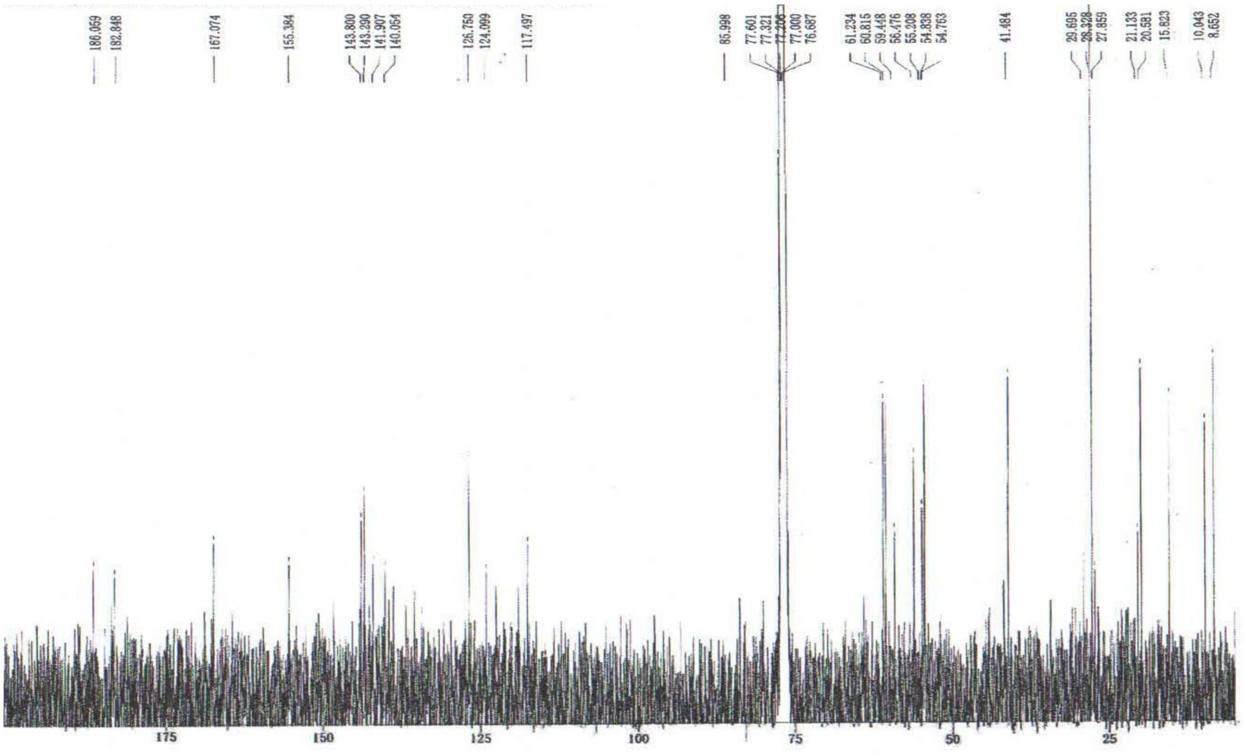

Figure S12. ^{13}C NMR (100 MHz) spectrum of **6a** in $CDCl_3$

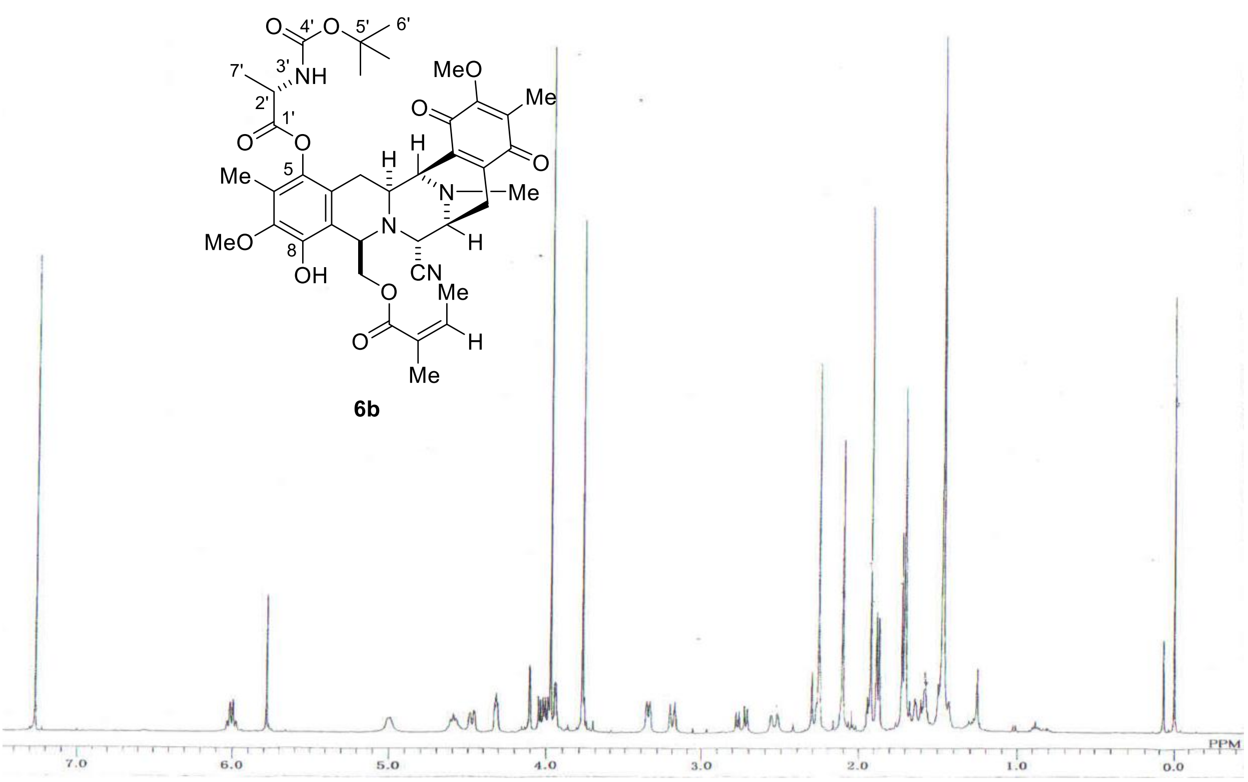

Figure S13. ^{1}H NMR (400 MHz) spectrum of **6b** in $CDCl_3$

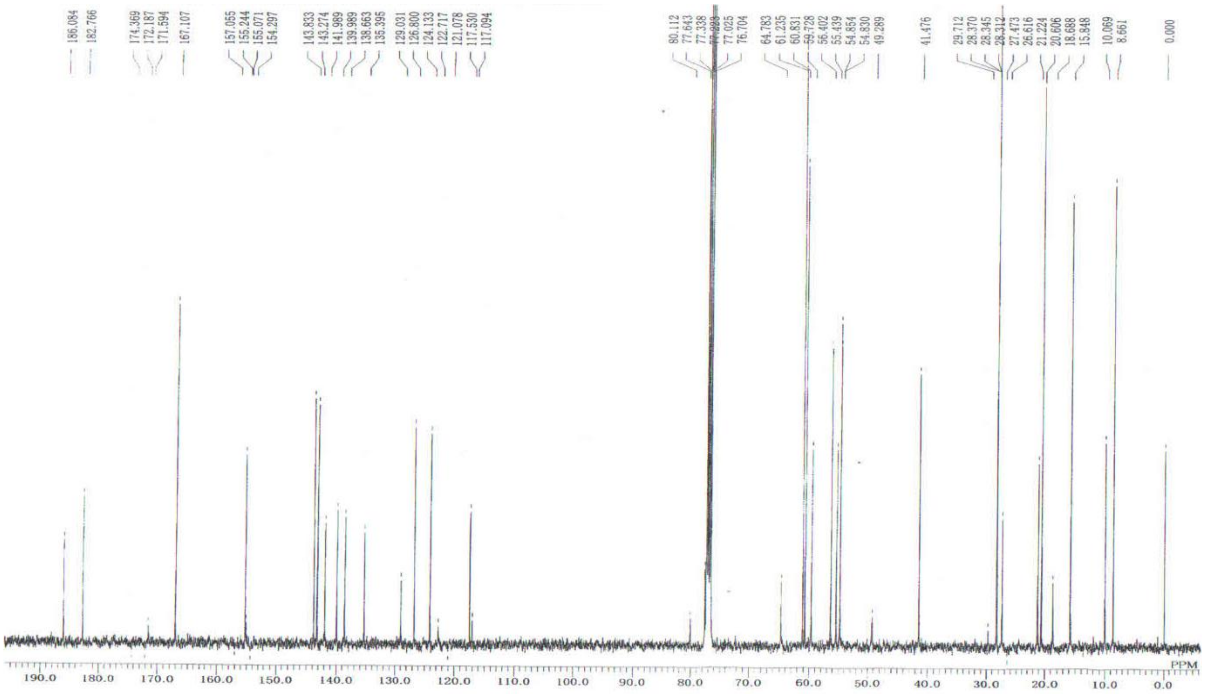

Figure S14. ^{13}C NMR (100 MHz) spectrum of **6b** in $CDCl_3$

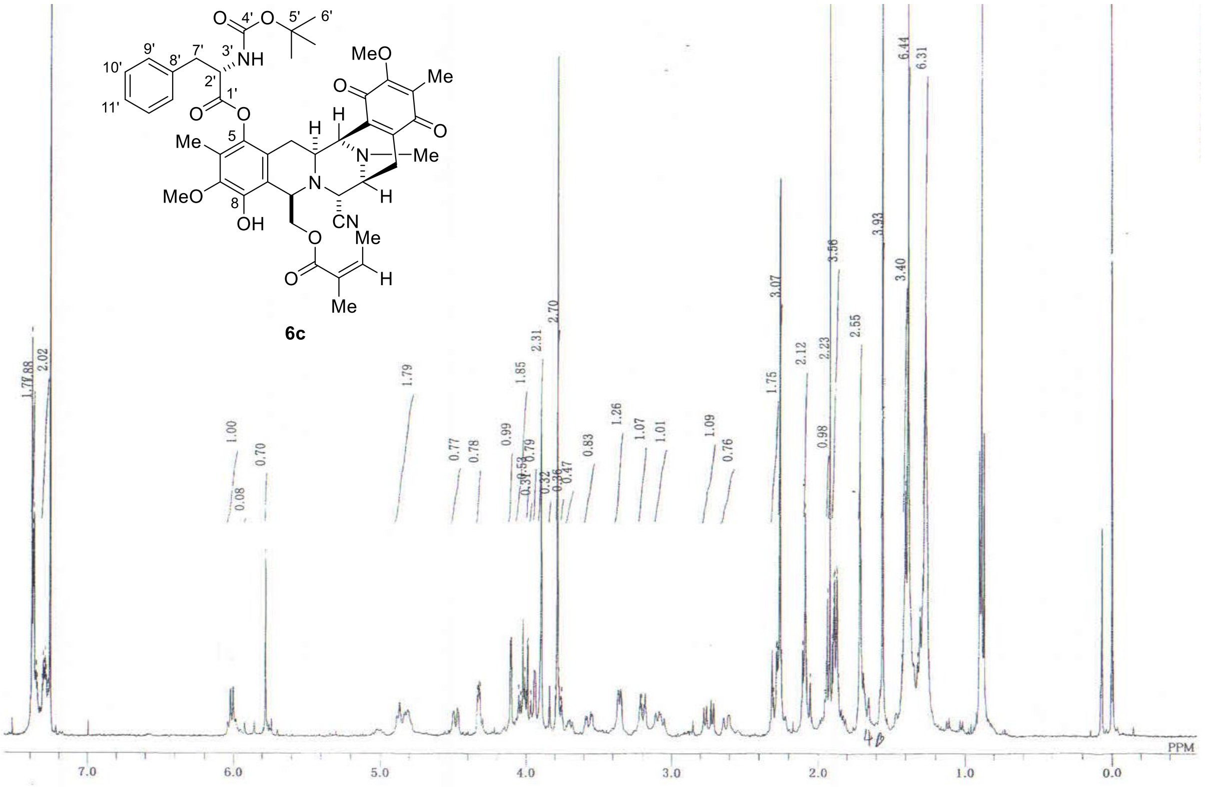

Figure S15. ^{1}H NMR (400 MHz) spectrum of **6c** in $CDCl_3$

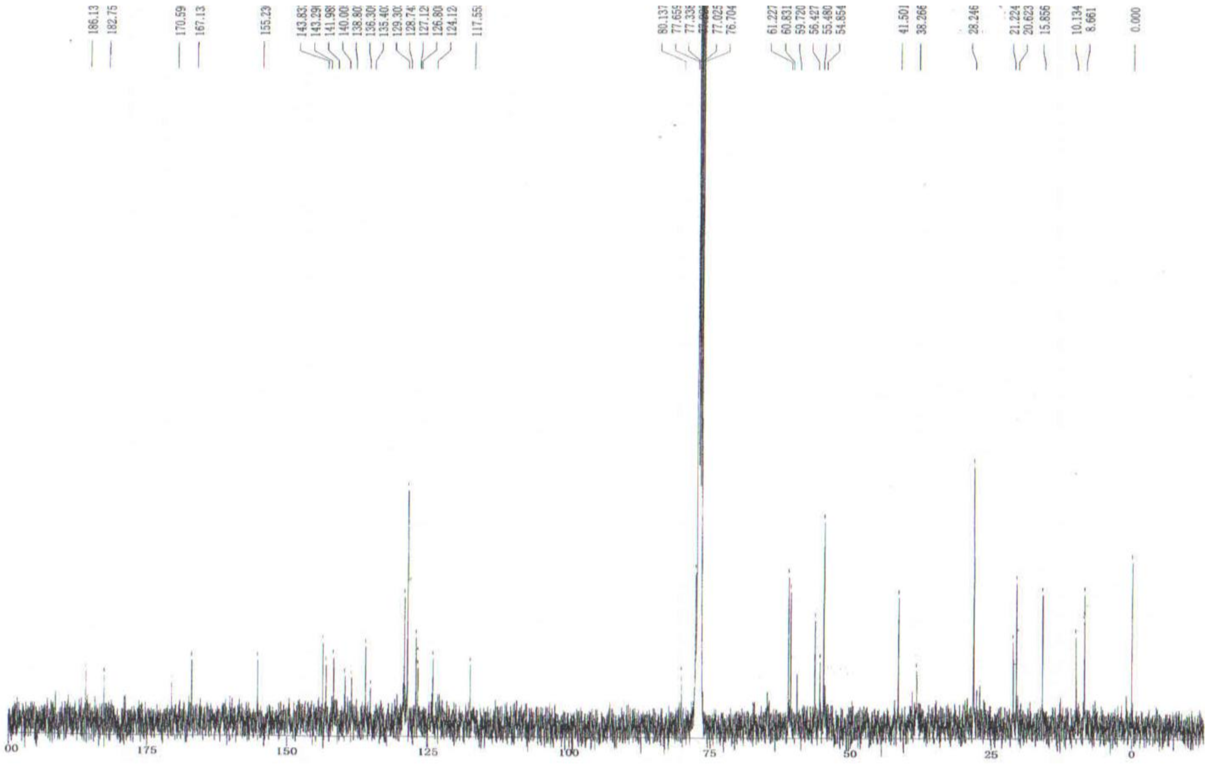

Figure S16. ^{13}C NMR (100 MHz) spectrum of **6c** in $CDCl_3$

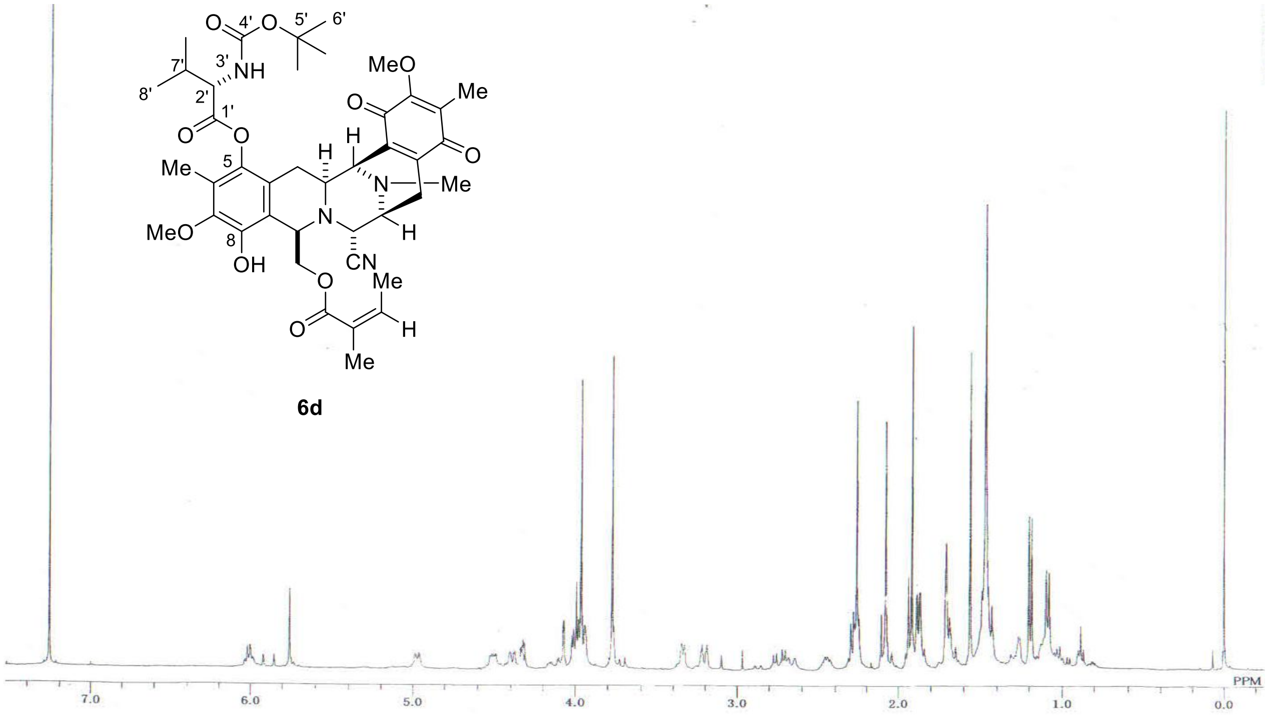

Figure S17. ^{1}H NMR (400 MHz) spectrum of **6d** in $CDCl_3$

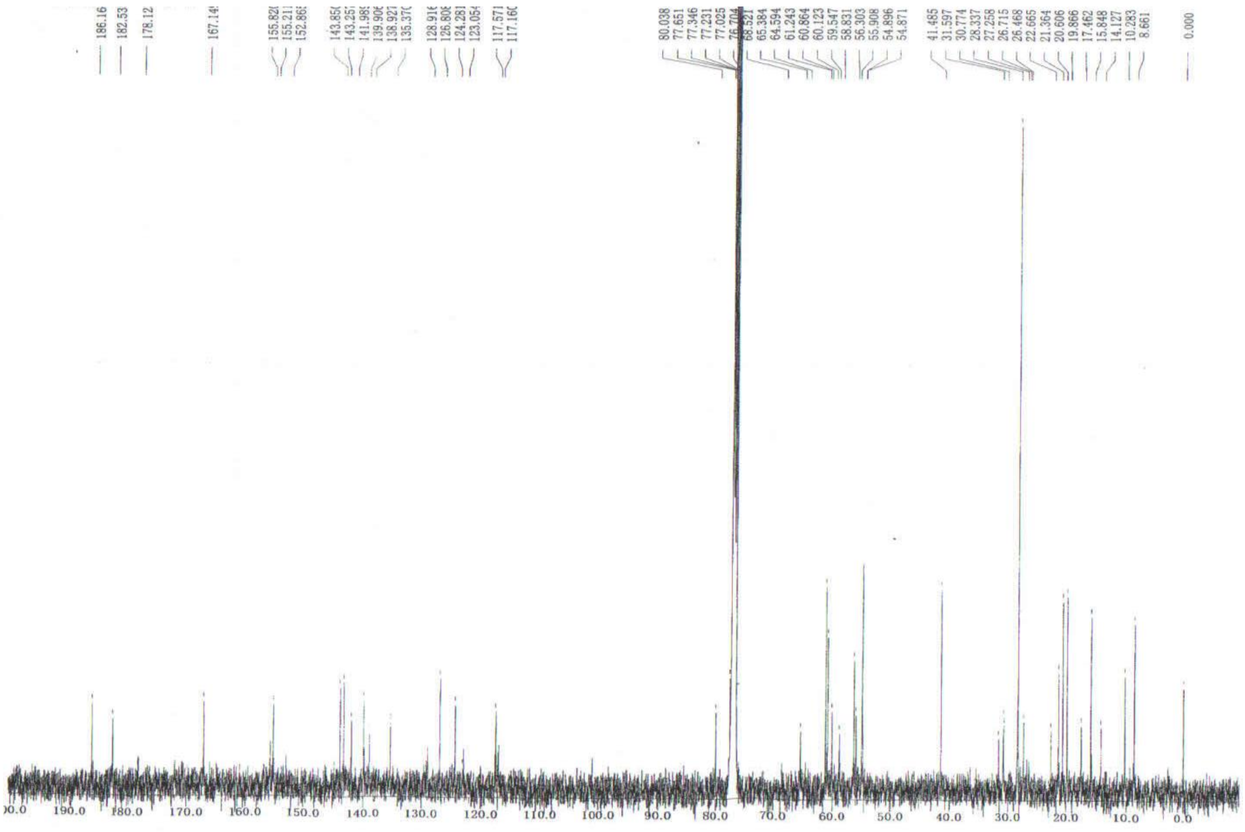

Figure S18. ^{13}C NMR (100 MHz) spectrum of **6d** in $CDCl_3$

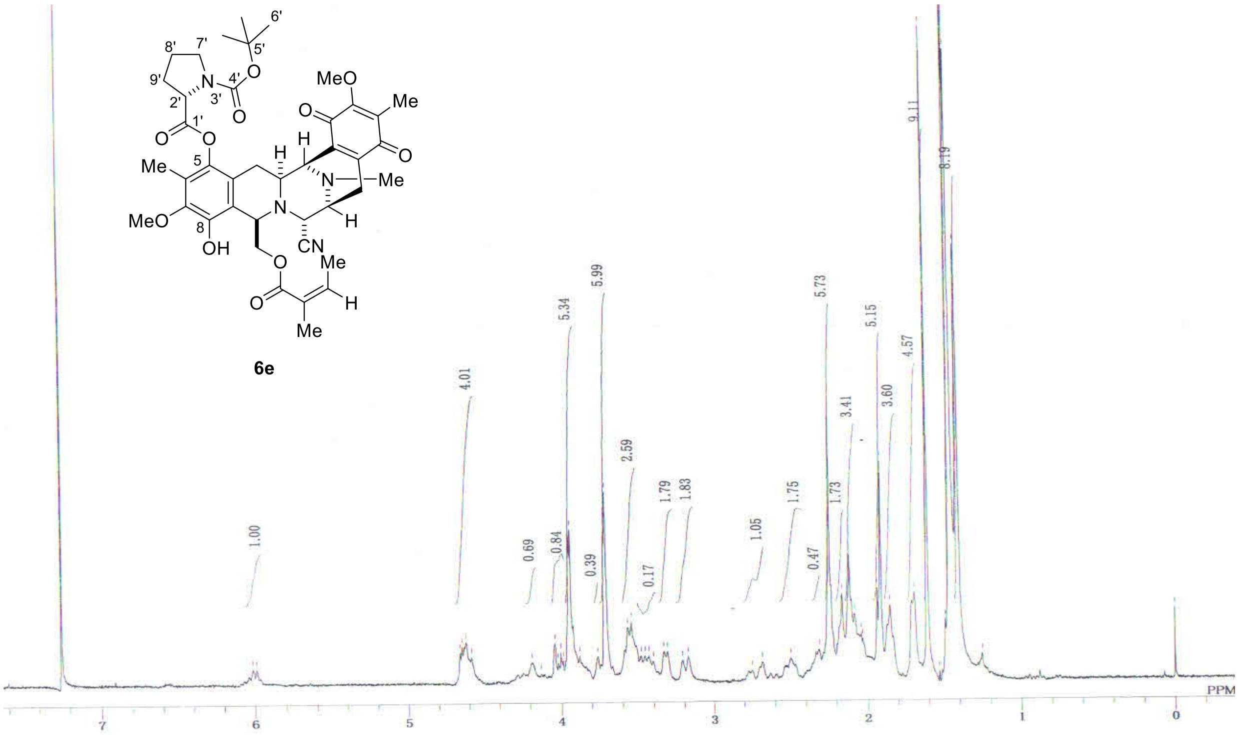

Figure S19. ^{1}H NMR (400 MHz) spectrum of **6e** in $CDCl_3$

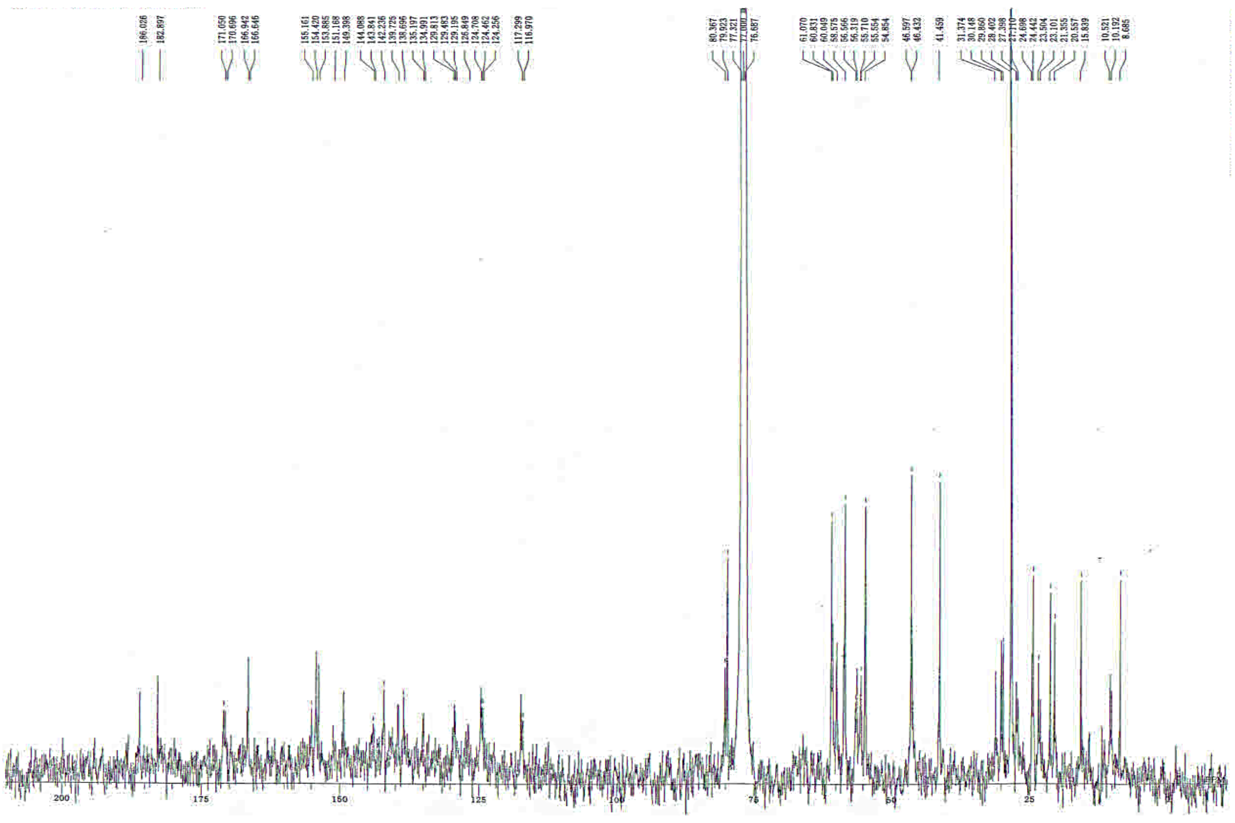

Figure S20. ^{13}C NMR (100 MHz) spectrum of **6e** in $CDCl_3$
